# Structural reorganization of SHP2 by oncogenic mutations and implications for oncoprotein resistance to allosteric inhibition

**DOI:** 10.1038/s41467-018-06823-9

**Published:** 2018-10-30

**Authors:** Jonathan R. LaRochelle, Michelle Fodor, Vidyasiri Vemulapalli, Morvarid Mohseni, Ping Wang, Travis Stams, Matthew J. LaMarche, Rajiv Chopra, Michael G. Acker, Stephen C. Blacklow

**Affiliations:** 1 Department of Biological Chemistry & Molecular Pharmacology, Harvard Medical School, Boston, MA 02115 USA; 2 Department of Cancer Biology, Dana-Farber Cancer Institute, Boston, MA 02215 USA; 3 Novartis Institutes for Biomedical Research, Cambridge, MA 02139 USA

## Abstract

Activating mutations in *PTPN11*, encoding the cytosolic protein tyrosine phosphatase SHP2, result in developmental disorders and act as oncogenic drivers in patients with hematologic cancers. The allosteric inhibitor SHP099 stabilizes the wild-type SHP2 enzyme in an autoinhibited conformation that is itself destabilized by oncogenic mutations. Here, we report the impact of the highly activated and most frequently observed mutation, E76K, on the structure of SHP2, and investigate the effect of E76K and other oncogenic mutations on allosteric inhibition by SHP099. SHP2^E76K^ adopts an open conformation but can be restored to the closed, autoinhibited conformation, near-identical to the unoccupied wild-type enzyme, when complexed with SHP099. SHP099 inhibitory activity against oncogenic SHP2 variants in vitro and in cells scales inversely with the activating strength of the mutation, indicating that either oncoselective or vastly more potent inhibitors will be necessary to suppress oncogenic signaling by the most strongly activating SHP2 mutations in cancer.

## Abstract (web summary)

Activating mutations of the non-receptor protein tyrosine phosphatase SHP2 can cause cancer. Here the authors present the crystal structure of SHP2^E76K^, the most frequent cancer-associated SHP2 mutation, which adopts an open-state structure and show that the allosteric inhibitor SHP099 can revert SHP2^E76K^ to its closed, autoinhibited conformation.

## Introduction

The non-receptor protein tyrosine phosphatase SHP2, encoded by the *PTPN11* proto-oncogene, is required for normal development and functions downstream of growth factor, cytokine, and adhesion receptors. SHP2 is ubiquitously expressed and required for sustained Ras-MAP kinase activation, and also modulates signaling through the NF-κB, JAK-STAT, PI3K-AKT, programmed cell death 1, and immune checkpoint (BTLA) pathways^1–4^. Activating mutations of SHP2 cause developmental disorders, such as Noonan Syndrome^5^, and occur frequently in patients with juvenile myelomonocytic leukemia (35%). Activating mutations are also observed recurrently in acute myeloid leukemia (5%)^6^, and at lower frequencies in other hematological cancers and solid tumors^4^. Cancer-associated mutations of SHP2 also cause leukemia in mice^7^, whereas genetic or chemical suppression of SHP2 has antitumor activity in a variety of cancer models^8,9^.

SHP2 contains two tandem SH2 domains (N-SH2 and C-SH2), a catalytic protein tyrosine phosphatase (PTP) domain, and a C-terminal tail that has at least two phosphorylation sites^10^. The X-ray structure of SHP2 reveals that in the basal state, this protein adopts a closed, autoinhibited conformation in which the N-SH2 domain engages the catalytic pocket of the PTP domain and sterically occludes the active site^11^. Normally, the binding of tyrosine-phosphorylated ligands to the SHP2 tandem SH2 domains is required to overcome autoinhibition^12^, but oncogenic mutations of SHP2 destabilize the autoinhibited conformation and lead to enhanced basal activity in the absence of tyrosine-phosphorylated ligand stimulation^13^.

Allosteric modulators that stabilize the closed form of SHP2 have been recently reported^14,15^. This class of allosteric inhibitors was designed to stabilize the autoinhibited state of the enzyme by acting as a molecular glue between the N-SH2 domain and the catalytic domain. One such compound, SHP099, binds to wild-type SHP2 with nanomolar affinity in biochemical assays, and exhibits antiproliferative activity in cancer cell lines that are dependent on receptor tyrosine kinases and wild-type SHP2^8^. It remains unclear, however, whether SHP2 activating mutations are amenable to allosteric inhibition by compounds such as SHP099, and if so, what range of mutations are susceptible.

Here, we investigate the impact of oncogenic mutations on the structure of SHP2 and on allosteric inhibition by SHP099 in biochemical and cellular assays. We report an open-state structure of a SHP2 variant that bears a potent activating mutation, E76K, which induces a dramatic domain reorganization to expose the active site and eliminates the binding pocket for the allosteric inhibitor SHP099. Although the E76K mutation reduces the inhibitory potency of SHP099 for SHP2 by more than 100-fold, binding of SHP099 to SHP2^E76K^ can revert the structure of this variant to its autoinhibited conformation. More generally, although a broad range of SHP2 oncogenic mutants can be inhibited by SHP099 in assays using the purified enzyme, the potency of inhibition scales inversely with the basal phosphatase activity of each variant, and in cells, the more active SHP2 oncoproteins display resistance to allosteric inhibition. These data show that oncoselective SHP2 inhibitors, or vastly more potent allosteric inhibitors, will be necessary to suppress the aberrant signaling that results from strongly activating SHP2 mutations in cancer.

## Results

### Structure of SHP2^E76K^ in an open conformation

**Fig. 1:**
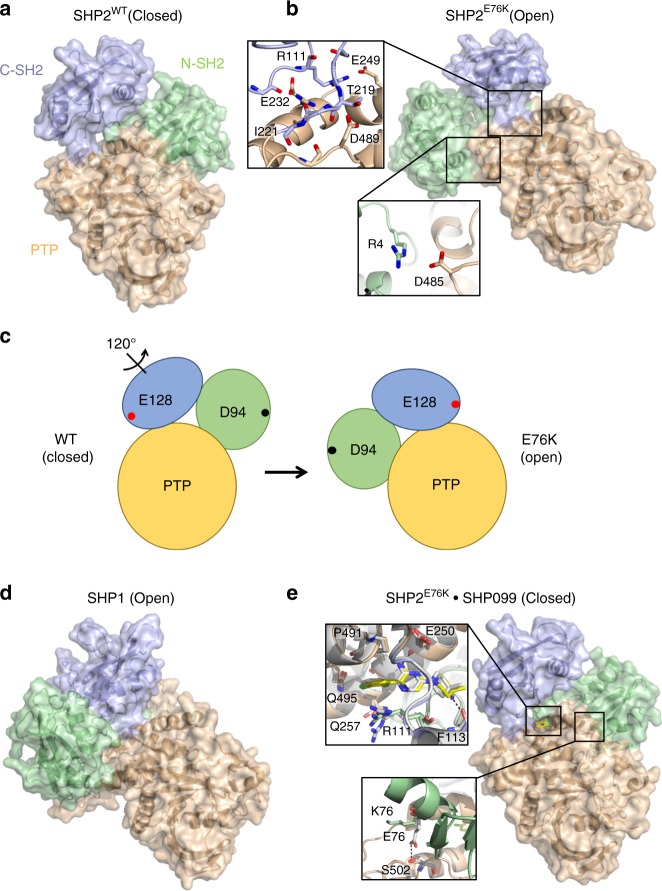
The open conformation of SHP2 E76K is closed by SHP099. **a** Basal structure of autoinhibited SHP2^WT^ (PDB:2SHP) with the N-SH2 domain displayed in green, C-SH2 in blue, and PTP in beige. **b** Structure of SHP2^E76K^ (1–525) reveals a 120**°** rotation of the C-SH2 domain, relocation of the N-SH2 domain to a PTP surface opposite the active site, and a solvent-exposed catalytic pocket. Insets show select N-SH2•PTP interdomain contacts and interactions between the C-SH2 and PTP domains. **c** Cartoon illustrating the domain movements that occur in SHP2^E76K^ upon adopting the open conformation. E128 and D94 are chosen as arbitrary reference points to illustrate the effect of the 120**°** rotation. **d** Open conformation of SHP1 (PDB:3PS5) is similar to that of SHP2^E76K^. **e** Interaction of SHP099 with SHP2^E76K^ rescues the autoinhibited conformation. Insets show select interactions of SHP099 with SHP2^E76K^ and SHP2^WT^, in addition to differential orientations of residue 76 in SHP2^E76K^ bound to SHP099 and in SHP2^WT^ bound to SHP099 (White, PDB:5EHR)

The autoinhibited conformation of SHP2 (PDB:2SHP) is stabilized by interactions between residues of the N-SH2 domain and regions of the phosphatase (PTP) domain that mask the catalytic pocket (Fig. 1a)^11^. Although amino acids of the SHP2 C-SH2 domain do not directly interact with either the N-SH2 or PTP domains, the orientation of the C-SH2 domain in the autoinhibited conformation of SHP2 is stabilized through interactions of the unstructured loop between N-SH2 and C-SH2 domains with N-SH2 and PTP domains and of the linker between C-SH2 and PTP domains with the PTP domain.

To investigate how the most frequently observed SHP2 oncogenic mutation, E76K, affects the structure of SHP2, we resolved the X-ray structure of near full-length SHP2^E76K^ (1–525) to 2.6 Å (Fig. 1b and Supplementary Table 1). We found that, in comparison with the basal conformation of SHP2^WT^, the C-SH2 domain of SHP2^E76K^ undergoes a 120-degree rotation with respect to the adjacent PTP domain, which fully exposes the PTP active site and repositions the N-SH2 domain onto a PTP domain surface opposite the catalytic pocket. The swiveling of the C-SH2 domain thus acts as a pivot to move the N-SH2 domain across the entire body of the PTP domain from one side to the other in switching from closed to open conformations (Fig. 1c). In both the basal SHP2^WT^ and open SHP2^E76K^ structures, the phosphotyrosyl binding site of each SH2 domain remains accessible to solvent.

The N-SH2 domain of SHP2, which is stabilized primarily through interdomain interactions in SHP2^WT^, is in part fixed by crystal contacts in SHP2^E76K^. Specifically, contacts between the αB helix and a symmetry-related PTP domain stabilize the position of the N-SH2 domain in SHP2^E76K^ in one of the two copies in the asymmetric unit. Nevertheless, there are interdomain contacts between the N-SH2 domain and the PTP domain that create a remarkably different N-SH2•PTP interdomain interaction surface in SHP2^E76K^ from the N-SH2•PTP interface observed in the autoinhibited conformation of SHP2^WT^. The buried surface area of the N-SH2 domain decreases from 1025 Å^2^ in SHP2^WT^ to 357 Å in E76K, with no overlapping contact residues. In SHP2^E76K^, the N-SH2 N-terminal residues and N terminus of the αB helix contribute to stabilizing the position of the N-SH2 domain at the alternative PTP surface. Specific interactions include a salt-bridge between R4 of the N-SH2 domain and D485 of the PTP domain, and hydrophobic contacts between T73 and V484.

The remodeled interface between the C-SH2 domain and the PTP domain in the open conformation is striking. Key residues that stabilize the position of the C-SH2 domain with respect to the PTP domain in the open-state structure of E76K lie in the segment preceding the C-SH2 domain core, and in the linker between the C-SH2 domain and the PTP domain. These residues include S109-R111, which contact E225, R229, and E232, and amino acids Q214 through I221 of the linker connecting the C-SH2 and PTP domains, which place several residues in backbone and side-chain contact with PTP domain residues D487 through D489, as well as with E249. One hydrogen bond, between T219 and D489, is conserved in both basal wild-type and E76K open conformations of SHP2.

Remarkably, the open conformation of SHP2^E76K^ also resembles the architecture of SHP1 (PDB:3PS5) in its open conformation (Fig. 1d)^16^. In the open state of SHP1, the C-SH2 domain also pivots 110-degrees relative to its position in the autoinhibited conformation of SHP1, which repositions the N-SH2 onto a surface of the SHP1 PTP domain similar in location to the N-SH2•PTP interface observed in the open conformation of SHP2^E76K^. In SHP1, however, the interactions between the N-SH2 and PTP domains are more extensive, burying 511 Å^2^ of surface area, which is substantially more than the 357 Å^2^ buried at this interface in the SHP2^E76K^ structure.

### SHP099 binding to SHP2^E76K^ restores the closed conformation

To determine whether SHP099 could revert SHP2^E76K^ to the closed, autoinhibited conformation, we determined the structure of SHP2^E76K^ bound to the small molecule inhibitor SHP099 to 2.8 Å (Supplementary Table I). Remarkably, when complexed with SHP099, SHP2^E76K^ adopts an autoinhibited conformation like that of SHP2^WT^, with near-identical C-SH2 and N-SH2 domain orientations relative to the PTP domain (Fig. 1e). The interactions between SHP099 and SHP2^E76K^ are similar to those seen in the structure of SHP099 bound to SHP2^WT^ (PDB: 5EHR). However, at the N-SH2•PTP interface, the K76 side chain is not in contact with the PTP surface and is instead poised to interact with Q79, which stabilizes the position of K76 in the open state of SHP2^E76K^. Together, the X-ray structures of unliganded and SHP099-bound SHP2^E76K^ show that the unoccupied enzyme adopts an open conformation that is similar to the open form of SHP1, and that binding of SHP099 reverts SHP2^E76K^ to a closed conformation that is near-identical to the autoinhibited state of SHP2^WT^.

**Fig. 2:**
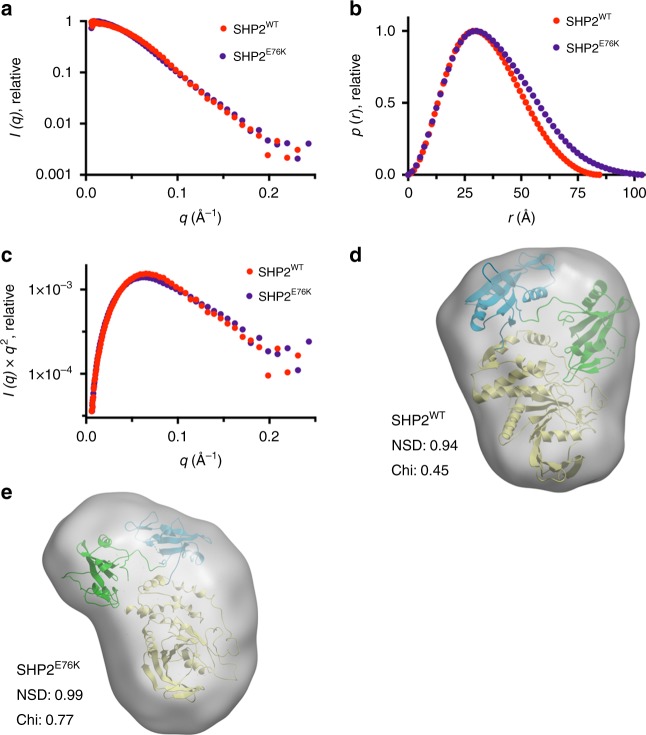
Small-angle X-ray scattering (SAXS) analysis of SHP2 proteins. **a** SHP2 protein SAXS intensity profiles across scattering vector *q*. SHP2^WT^ is shown in red and SHP2^E76K^ in violet. **b** p(r) functions of SHP2^WT^ and SHP2^E76K^. **c** Kratky analysis of SAXS data. **d** Fitting of the X-ray structure of SHP2^WT^ (PDB:2SHP) into the SAXS envelope of SHP2^WT^. **e** Fitting of the X-ray structure of SHP2^E76K^ into the SAXS envelope

To test whether the X-ray structure of SHP2^E76K^ in the crystal is representative of its conformation in solution, we performed small-angle X-ray scattering (SAXS) analysis of SHP2^E76K^ and compared it with SAXS data acquired for SHP2^WT^. Fitting of the background-subtracted scattering profiles of each protein (Fig. 2a) to the pair distance distribution p(r) function revealed that both proteins adopt compact conformations, with radii of gyration values (*R*_g_) of 26.2 ± 0.2 Å for SHP2^WT^ and 29.2 ± 0.3 Å for SHP2^E76K^ (Fig. 2b and Supplementary Table 2). The tail of the SHP2^E76K^ p(r) function displayed a shallower slope than that of SHP2^WT^, resulting in a calculated maximal end-to-end distance (*D*_max_) of 100 ± 4 Å, whereas the *D*_max_ for SHP2^WT^ was 83 ± 3 Å. Kratky analysis of the SAXS data for both proteins indicates that they are both well-folded and monodisperse in solution (Fig. 2c). To assess whether the SAXS-derived particle dimensions of SHP2^E76K^ are compatible with the conformation of SHP2^E76K^ seen in the X-ray structure, we compared SAXS envelopes of SHP2^WT^ and SHP2^E76K^ with their respective X-ray structures. The SAXS-determined SHP2^WT^ particle envelope agrees with the X-ray structure of SHP2^WT^ in its closed conformation (PDB:2SHP), with normalized spatial discrepancy (NSD) and chi values of 0.94 and 0.45, respectively (Fig. 2d). Likewise, the X-ray structure of SHP2^E76K^ fits the SHP2^E76K^ SAXS envelope particularly well (NSD 0.99, Chi 0.77; Fig. 2e). Taken together, these results provide additional evidence that SHP2^E76K^ adopts a solution conformation consistent with the open conformation seen crystallographically.

### SHP2 oncoprotein sensitivity to allosteric modulators

**Fig. 3:**
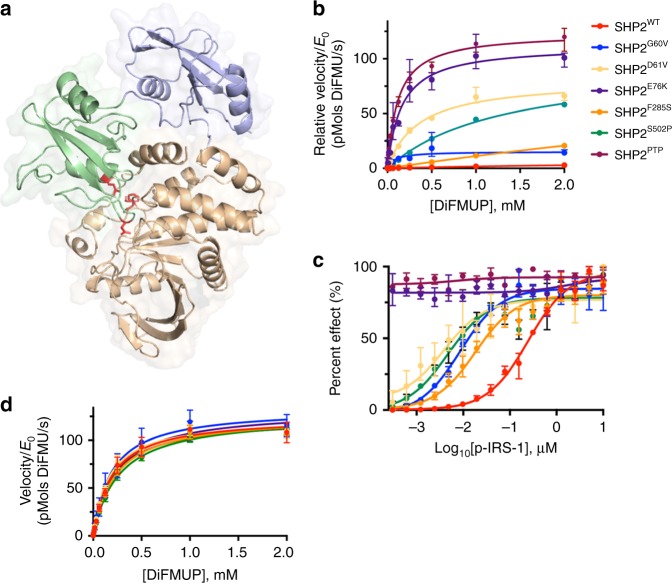
SHP2 cancer mutations enhance basal activity and response to phospholigands. **a** Sites of oncogenic mutations of SHP2 mapped onto the autoinhibited structure of SHP2 (PDB: 2SHP), which localize to the N-SH2:PTP interdomain interface. The N-SH2 domain of SHP2 is displayed in blue, C-SH2 in green, PTP in beige, and cancer mutations in red. **b** Normalized basal activity of SHP2^WT^ and cancer mutants (5 nM WT, 1 nM F285S, 0.2 nM G60V, 0.1 nM D61V & S502P, and 0.05 nM E76K & PTP), plotted as a function of DiFMUP concentration. **c** Enzymatic activity of SHP2 analogs (0.05 nM) in the presence of DiFMUP (200 μM) plotted as a function of the concentration of a synthetic IRS-1-derived bisphosphorylated peptide (p-IRS-1, SLNY(p)IDLDLVK-dPEG_8_-LSTY(p)ASINFQK). **d** Enzymatic activity of various SHP2 proteins (0.05 nM) plotted as a function of DiFMUP concentration in the presence of saturating (10 μM) p-IRS-1. Data points are presented as mean ± standard deviation (*n* = 3)

To investigate the biochemical effects of oncogenic mutations of SHP2 on allosteric activation and inhibition, we purified a series of cancer-associated SHP2 mutants that exhibit a spectrum of basal phosphatase activity. The oncogenic mutations typically reside in the N-SH2 or PTP domains of the phosphatase, and map almost exclusively to the interface between these domains in the autoinhibited structure of SHP2 (Fig. 3a). Using the fluorogenic substrate 6,8-Difluoro-4-Methylumbelliferyl Phosphate (DiFMUP), the basal activity of the SHP2 variants investigated here increases from SHP2^WT^ < SHP2^F285S^ < SHP2^G60V^ < SHP2^S502P^ < SHP2^E76K^, where SHP2^E76K^ displays activity similar to the isolated PTP domain (SHP2^PTP^, Fig. 3b). To explore how SHP2 cancer mutations influence stimulation of the phosphatase by phospholigands, we titrated SHP2 variants with a synthetic bisphosphorylated peptide derived from IRS-1 (p-IRS-1), which stimulates phosphatase activity by engaging both the N-SH2 and C-SH2 domains, and quantified the effect on DiFMUP dephosphorylation as a function of added p-IRS-1 concentration (Fig. 3c). This analysis shows that the oncogenic mutations of SHP2 tested here do not detectably alter the maximal catalytic activity of the protein as observed in the presence of a saturating concentration of p-IRS-1, but rather increase both basal phosphatase activity and the sensitivity to phospholigand stimulation (Fig. 3d). Specifically, the cancer-associated SHP2 mutants reduce the concentration of p-IRS-1 required to stimulate the phosphatase activity of SHP2 by one to two orders of magnitude (the p-IRS-1 AC_50_ value for stimulation), except in the case of the most active mutant SHP2^E76K^, which is essentially fully activated in the absence of p-IRS-1 and thus insensitive to further stimulation (Fig. 3d).

**Fig. 4:**
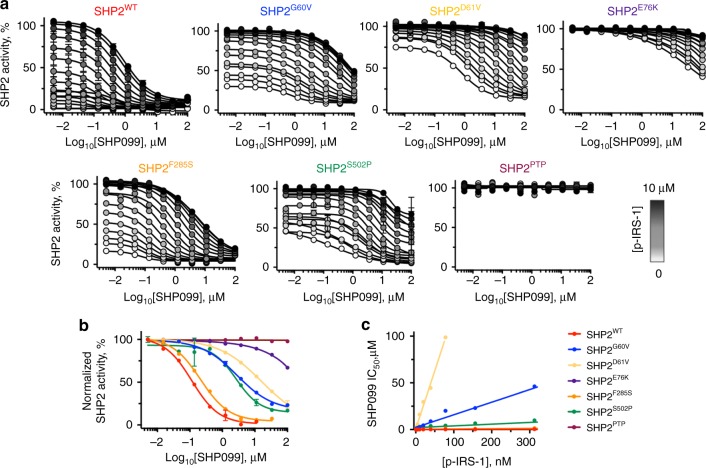
Allosteric inhibition of SHP2 is influenced by cancer mutations and activating ligands in vitro. **a** Analysis of the effect of p-IRS-1 on the effectiveness of SHP099 inhibition of DiFMUP dephosphorylation by wild-type and mutant SHP2 proteins. **b** SHP099 dose–response curves for inhibition of DiFMUP dephosphorylation by wild-type and oncogenic SHP2 proteins (0.1 nM) in the presence of a fixed concentration of p-IRS-1 (10 nM). **c** SHP099 IC_50_ values plotted as a function of p-IRS-1 peptide concentration for wild-type SHP2 protein (red) and for a range of different oncogenic SHP2 mutants. Data points are presented as mean ± standard deviation (*n* = 2)

To investigate how the various oncogenic SHP2 mutations impact allosteric inhibition of the phosphatase by SHP099, we titrated SHP099 against SHP2 proteins in the presence and absence of p-IRS-1 and quantified the phosphatase activity of the different enzymes using DiFMUP (Fig. 4a, b). We found that under basal conditions, SHP2 cancer mutations weaken the observed potency of SHP099 when compared with SHP2^WT^, and that the inhibitory potency of SHP099 generally scales inversely with the basal phosphatase activity of the cancer mutant (Fig. 4 and Supplementary Table 3). For the most active mutant SHP2^E76K^, we extended the dose–response curve to a higher SHP099 concentration in order to deduce its IC50 of 34 ± 6 μM in the absence of p-IRS-1 (Supplementary Fig. 1). As anticipated, the presence of added p-IRS-1 decreases the inhibitory potency of SHP099 for all forms of SHP2, with the relationship between p-IRS-1 and SHP099 fitting a competitive binding model (Fig. 4c). The IC_50_ of SHP099 for SHP2^WT^, for example, increases from 92 nM (95% CI: 60–142 nM) at 2 nM p-IRS-1 peptide (Supplementary Fig. 2) to 1.7 μM (at a saturating concentration of 10 μM p-IRS-1 peptide). Other SHP2 cancer mutants show a similar trend in response to p-IRS-1 peptide (Fig. 4c and Supplementary Table 3). These data establish that inhibition of SHP2 by SHP099 is antagonized by both activating mutations and by phospholigands, which when combined can result in substantial losses of SHP099 inhibitory potency.

### Sensitivity of SHP2 signaling to SHP099 in cellular assays

**Fig. 5:**
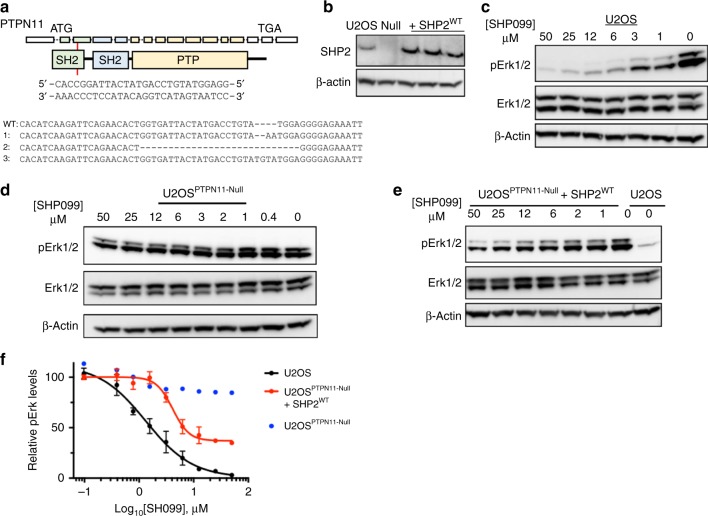
Establishment of PTPN11-null U2OS (U2OS^PTPN11−/−/−^) cells for SHP2 rescue experiments. **a** CRISPR guide, shown 5′-to-3′, designed to target exon 3 of PTPN11. PTPN11 is displayed as 16 exons, which correspondingly encode N-SH2 (green), C-SH2 (blue), and PTP (beige) domains. Allele sequences of U2OS^PTPN11−/−/−^ (null) cells are compared with the sequence of wild-type SHP2. **b** Western blot showing SHP2 expression levels in wild-type U2OS cells (U2OS), PTPN11-null cells (null), and null cells transfected with full-length SHP2^WT^. **c** Western blot analysis showing SHP099 reduces pErk levels in wild-type U2OS cells. **d** Western blot analysis showing SHP099 does not reduce pErk levels in null cells. **e** Western blot analysis showing pErk levels in null cells transfected with SHP2^WT^ are sensitive to SHP099. **f** Densitometry quantification of SHP099-dependent pErk reductions, in wild-type U2OS and PTPN11-null cells re-expressing SHP2^WT^, derived from the experiments shown in panels **c**–**e**. The left-most data point of each graph refers to pErk levels at 0 μM SHP099 in each respective cell line, which was used to normalize data in the quantification analysis. Data points are presented as mean ± standard error of the mean

**Fig. 6:**
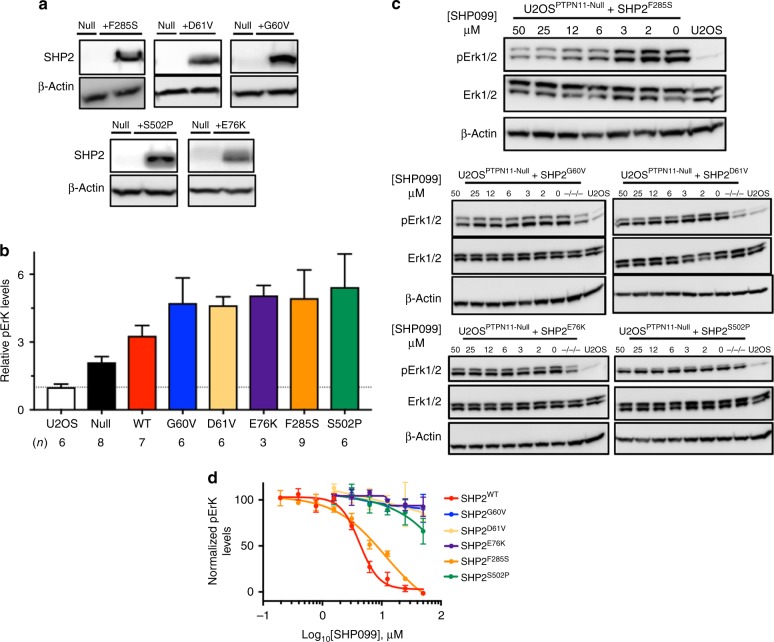
Oncogenic mutants of SHP2 negatively impact allosteric inhibition in SHP2-deficient U2OS cells. **a** Western blot quantification of SHP2 expression levels in wild-type U2OS cells (U2OS) and in SHP2-deficient U2OS cells (PTPN11-null) in the presence and absence of expression of SHP2 oncogenic variants. **b** Western blot analysis showing relative pErk levels in wild-type, SHP2-deficient, and SHP2-rescued U2OS cells. **c** Western blot determination of pErk levels in SHP2-deficient U2OS cells, in the presence and absence of the SHP2 allosteric inhibitor SHP099. **d** Quantification of pErk levels in SHP2-deficient U2OS cells expressing SHP2^WT^ and oncogenic mutants upon titration of SHP099. Data points are presented as mean ± standard error of the mean

Having defined the effects of oncogenic SHP2 mutations on SHP099 allosteric inhibition of the purified proteins, we next sought to assess the sensitivity of this spectrum of cancer mutants to modulation of signaling by SHP099 in cells. For this purpose, we prepared a SHP2-deficient cell line using the CRISPR-Cas9 system (Fig. 5a, b), and then added either SHP2^WT^ or different cancer variants for evaluation, measuring phosphorylated-Erk (pErk) levels as a readout of SHP099 inhibitory activity. We performed the knockout in U2OS cells, because this cell line induces pERK in response to EGF stimulation, which is attenuated in the presence of SHP099 (Supplementary Fig. 3), and because U2OS cells can tolerate SHP2 knockout. Although SHP099 treatment dramatically reduces pErk levels of wild-type U2OS cells, as assessed by western blot (Fig. 5c), pErk levels in *PTPN11*-null U2OS cells are unaffected by SHP099 treatment (Fig. 5d). Reintroduction of full-length SHP2^WT^ into *PTPN11*-null U2OS cells by transfection results in expression of SHP2 in amounts about five-fold greater than in parental U2OS cells, as assessed by western blot (Fig. 5b). In *PTPN11*-null U2OS cells that re-express SHP2^WT^, pErk sensitivity to SHP099 is partially restored (Fig. 5e; we attribute the incomplete ~ 65% reduction of pErk levels by SHP099 in SHP2^WT^-rescued cells to a rewiring of SHP2 signaling that occurs upon SHP2 knockout, as evidenced by increased basal pErk levels in the null cells compared with the parental line). Quantification of the western blot data gives an IC_50_ value for pErk reduction by SHP099 in the *PTPN11*-null cells re-expressing SHP2^WT^ (4 ± 2 μM) in line with the IC_50_ observed for wild-type U2OS cells (2 ± 1 μM; Fig. 5f). We then probed the effects of oncogenic SHP2 mutants on the sensitivity to SHP099 in the same assay. SHP2 cancer mutants (SHP2^G60V^, SHP2^D61V^, SHP2^E76K^, SHP2^F285S^, and SHP2^S502P^) introduced into *PTPN11*-null U2OS cells were expressed in amounts comparable to the reintroduced SHP2^WT^ in the SHP2-null cells (Fig. 6a). The expression of these oncogenic variants in *PTPN11*-null U2OS cells is associated with pErk levels approximately five-fold greater than in parental U2OS cells (Fig. 6b), indicating that these mutants strongly stimulate this pathway when transfected into the null cell line. SHP099 treatment reduces pErk levels in U2OS cells harboring the weakly stimulating SHP2^F285S^ enzyme (Fig. 6c), with an IC_50_ for pErk reduction approximately four-fold greater (8 ± 2 μM) than in SHP2^WT^ cells (Fig. 6d; see Supplementary Figs. 5–15 for full lanes associated with western blot data from Figs. 5, 6); in contrast, SHP2^S502P^ displays little, if any, loss of pErk at 50 μM SHP099, and SHP099 does not significantly inhibit Erk phosphorylation in the other cancer mutants investigated. We also enforced expression of wild-type and oncogenic forms of SHP2 in the parental U2OS cells and observed qualitatively similar findings (Supplementary Fig. 4). These results indicate that oncoproteins with weakly activating mutations, such as F285S, remain sensitive to enzyme inhibition in cells, whereas more strongly activating mutations are effectively resistant to allosteric inhibition by SHP099.

## Discussion

There is great interest not only in understanding how frequently observed cancer mutations in oncogenes and tumor suppressors impact protein structure and function, but also in deducing how such mutations affect therapies that target the wild-type protein. Here, we have pursued a mechanistic understanding of how activating mutations affect the structure of the protein phosphatase SHP2 and its sensitivity to the wild-type directed allosteric inhibitor SHP099. Strikingly, the most frequently occurring SHP2 cancer mutation, a potent E76K substitution also found in Noonan’s syndrome, favors an open conformation that allows free access of substrates to the active site, in contrast to the autoinhibited conformation favored by SHP2^WT^ in its basal state, providing a structural framework for understanding how this class of gain-of-function mutants, associated with diseases such as Noonan’s syndrome and cancer, lead to increased phosphatase activity. The open state of SHP2^E76K^ involves a 120-degree pivot of the C-SH2 domain, relocalization of the N-SH2 domain to an alternative PTP domain interaction surface, and full exposure of the phosphatase active site. The contacts that stabilize this open C-SH2 domain orientation suggest that, in the absence of critical interactions at the N-SH2•PTP autoinhibition interface, this position is the preferred orientation of the C-SH2 domain, and likely also represents an accessible open conformation of wild-type SHP2 upon activation by phosphotyrosine-containing peptide sequences, as opposed to a neomorphic conformation resulting from the oncogenic E76K mutation. The discrete conformation of SHP2^E76K^ in its open state was unexpected, but the remarkable agreement between the SHP2^E76K^ crystal structure, SAXS-determined solution particle dimensions, and the structure of the related phosphatase SHP1 in its open state further underscore the relevance of the open state structure.

Recently, allosteric inhibitors of SHP2 have been discovered that biochemically suppress phosphatase activation in the presence of stimulatory phospholigands and weakly activating cancer mutations. Although such molecules have been demonstrated to reduce the viability of RTK-driven tumor cells through inhibition of MAPK signaling in cells that have normal SHP2 alleles, we lack an understanding of whether SHP2 mutations observed in patients with *PTPN11*-mutated malignancies can be effectively targeted by currently available allosteric inhibitors.

Strikingly, binding of the SHP2 allosteric inhibitor SHP099 to SHP2^E76K^ can revert SHP2^E76K^ to the autoinhibited conformation seen in the basal state of SHP2^WT^. We found, however, that the efficacy and potency of SHP099 inhibition decreases as the amount of stimulatory phospholigand or as the strength of the activating mutation increases. SHP099 inhibition of the most active oncogenic SHP2 mutants investigated here, SHP2^D61V^ and SHP2^E76K^, is orders of magnitude weaker than inhibition of SHP2^WT^, and the responsiveness of the mutants tested using purified protein is mirrored in cell lines engineered to express the same spectrum of mutated SHP2 proteins. Thus, although it is possible to revert the most potent activating mutation back to the closed conformation with SHP099 in vitro, cell lines expressing strongly activating mutations are effectively resistant to SHP099, likely because stabilization of the open conformation by phosphotyrosine ligands further increases the favorability of the open state in cells.

Because nearly half of patients with *PTPN11*-mutated cancers bear strongly activating mutations of E76, D61, G60, and S502, an allosteric inhibitor would need to exhibit at least two additional orders of magnitude greater potency than SHP099 and a longer target residence time to achieve successful suppression of SHP2 activity of tumors bearing these mutations in a clinical setting^17^. Whereas strongly activating mutations of SHP2 confer resistance to allosteric inhibition, orthosteric SHP2 inhibitors, in principle, may exhibit more effective oncogene selectivity because their mechanism of action targets the open conformation favored by cancer-associated mutations. The primary challenge with orthosteric inhibitors is to achieve sufficient selectivity for SHP2 over other cellular phosphatases, which may be further thwarted by the SHP2 cancer mutations that localize to residues near the active site, such as S502, G503, and Q510. One orthosteric inhibitor, called 11-a 1, is reported to have fivefold or greater selectivity for SHP2 over related protein tyrosine phosphatases and to show promise in a melanoma model^18–20^. However, another recent study has called into question the specificity of such active site-directed inhibitors in the cellular context^21^. A combination of potent allosteric inhibitors that target distinct pockets may be yet another alternative strategy for inhibiting active SHP2 oncogenic mutants^22^. Finally, the open state structure suggests an experimental path for discovery of allosteric or active-state modulators that stabilize conformations of SHP2 other than its basal, autoinhibited state in strongly activated mutants.

## Methods

### Materials

DiFMUP (D22065) and 6,8-difluoro-7-hydroxy-4-methylcoumarin (DiFMU; D6566) were purchased from LifeTechnologies, Tris(2-carboxyethyl)phosphine hydrochloride from Pierce^TM^ (20490), potassium bisperoxo(1,10-phenanthroline) oxovanadate (bpV(Phen)) from Enzo Life Sciences (ALX-270-204) and phosphorylated peptides from AnaSpec. Unless otherwise stated, all reagents were obtained from commercial sources and used without further purification.

### SAXS

Size-exclusion chromatography (SEC)–SAXS experiments were performed at BioCAT (beamline 18-ID; Advanced Photon Source, Argonne National Laboratory)^23^. The setup included a focused 12-keV (1.03 Å) X-ray beam, a 1.5-mm quartz capillary sample cell, a sample-to-detector distance of ∼ 2.5 m, and a Mar165 charge-coupled-device detector. The momentum transfer (scattering vector) *q* was sampled from ∼ 0.0065 to 0.3 Å^−1^. In order to ensure sample monodispersity, we used an in-line SEC setup, which included an Äkta pure fast-performance liquid-chromatography unit and a Superdex200 10/300 GL column (GE Healthcare Life Sciences). The column outlet was directly connected to the SAXS sample cell. One-second exposures were collected every 5 s during the gel filtration chromatography run. Exposures before and after the elution of the sample were averaged and used as the buffer curve, and the exposures during elution (coincident with the UV peak on the chromatogram) were treated as the protein-buffer curves. Data were corrected for background scattering by subtracting the buffer curve from the protein-buffer curves. Each peak fraction frame was analyzed using the PRIMUS, GNOM, DAMMIF, DAMMIN, CRYSOL, DAMAVER, and SUPCOMB programs of the ATSAS package^24^ (Molecular envelopes were prepared by averaging 20 bead models generated by DAMMIF with DAMAVER, for use as a DAMMIN starting model). Crystal structure models were fitted to the envelope using SUPCOMB, and resulting fits were compared with the original subtracted data using CRYSOL.

### Quantification of basal SHP2 phosphatase activity

SHP2 proteins were expressed and basal activity was measured as described^13^. In brief, 2.5 nM SHP2^WT^, 0.5 nM SHP2^F285S^, 0.1 nM SHP2^G60V^, 0.05 nM SHP2^S502P^ and SHP2^D61V^, and 0.025 nM SHP2^E76K^ and SHP2^PTP^ were prepared in 60 mM Hepes pH 7.2, 75 mM KCl, 75 mM NaCl, 1 mM ethylenediaminetetraacetic acid (EDTA), 0.05% Tween-20, and 1 mM tris(2-carboxyethyl)phosphine (TCEP) (buffer 1), and added to a linear titration of DiFMUP (4 mM to 1 fM). DiFMUP dephosphorylation was measured at ex. 358, em. 450 nm on a Spectramax M5, and raw velocity data was converted to product 6,8-difluoro-7-hydroxy-4-methylcoumarin (DIFMU)/time through preparing a standard curve of DiFMU/DiFMUP. To allow direct comparison between enzyme trajectories, velocity data were normalized to enzyme concentration to yield relative velocity, which was then fit to the following substrate inhibition equation (Eq. (1)) to extrapolate kinetic parameters:

1 $$V=V_{{\mathrm{max}}} \cdot \left[ {{S}} \right]/\left( {{{K}}_{\mathrm{M}} + \left[ {{S}} \right]\left( {1 + \left[ {{S}} \right]/{{K}}_{{i}}} \right)} \right)$$

Where *V* is enzyme velocity, *V*_max_ is maximal enzyme activity, *K*_M_ is the Michaelis–Menten constant, [*S*] is substrate concentration, and *K*_*i*_ is the substrate dissociation constant. In vitro SHP2 phosphatase assays were performed in triplicate on the same day.

### SHP2 phosphatase activity upon p-IRS-1 peptide stimulation

Wild-type and mutant SHP2 proteins were diluted to 0.1 nM and treated with either varying concentrations of diphosphorylated IRS-1 peptide (pY1172-Peg-PY1222, SLNY(p)IDLDLVK-dPEG_8_-LSTY(p)ASINFQK), or for maximal activation, 10 μM pY1172-Peg-PY1222. Phosphatase activity was measured through the dephosphorylation DiFMUP (200 μM, ex. 385, em. 450 nm) on a Spectramax M5 plate reader and converted to product (DiFMU)/time by preparing a standard curve of DiFMU/DiFMUP. Velocity data were fit to the following three-parameter dose–response curve (Eq. (2)) to extrapolate EC_50_ values:

2 $${V} = {\mathrm{Basal}} + \left( {{\mathrm{Max}} - {\mathrm{Basal}}} \right)/\left( {1 + 10^{\left( {{\mathrm{LogEC}}_{50} - \left[ {{S}} \right]} \right)}} \right)$$

Where *V* represents enzyme velocity, Basal is basal response, Max is maximal response, [*S*] is substrate concentration, and EC_50_ is the substrate concentration that yields response half way between basal and maximal.

### The influence of SHP2 cancer mutations on SHP099 efficacy

Wild-type and mutant SHP2 proteins were diluted to 0.1 nM in buffer 1, in the presence of SHP099, ranging in concentration from 100 μM to 5 nM. To which, bis-tyrosylphosphorylated peptide pY1172-Peg-PY1222 was either added at 10 nM or titrated, from 10 μM to 1 nM, followed by 100 μM DiFMUP. The reaction was then allowed to proceed for 30 minutes at room temperature, quenched with bpV(Phen) and assayed on a PerkinElmer EnVision^TM^ plate reader. For visualization, raw data were converted to percent effect (Eq. (3)) and fit to a variable slope four-parameter inhibitor response curve (Eq. (4)) to extrapolate SHP099 IC_50_ values.

3 $${\mathrm{Percent}}\,{\mathrm{Effect}} = \left( {{\mathit{x}} - {\mathrm{min}}} \right)/\left( {\max - {\mathrm{min}}} \right) \times 100\%$$

Where *x* is the sample value (RFU), min is the RFU value observed for SHP2^WT^ in the presence of 100 μM SHP099 and absence of pY1172-Peg-PY1222, after reacting with 100 μM DiFMUP for 30 min at room temperature, and max is the maximum RFU value observed for each respective SHP2 protein in the absence of SHP099 and presence of 10 μM pY1172-Peg-PY1222, after reacting with 100 μM DiFMUP for 30 min at room temperature.

4 $${\mathrm{Percent}}\,{\mathrm{Effect = min}} + \left( {{\mathrm{max}} - {\mathrm{min}}} \right)/\left( {1{\mathrm{ + }}10^{\left( {{\mathrm{logIC}}_{50} - {\mathit{x}}} \right) \times {\mathrm{hillslope}}}} \right)$$

Where max and min are the respective maximum and minimum values of each dose–response curve, IC_50_ is the SHP099 concentration that gives response half way between max and min, and hillslope describes the steepness of each curve. In vitro SHP099 dose responses were performed in high throughput, with two technical replicates, on the same day.

### Development of SHP2-null U2OS cells

*PTPN11*−/−/− U2OS cells were generated using the methodology described by Zhang et al.^25^. In brief, CRISPR guides against the first three exons of the *PTPN11* proto-oncogene were designed using the Sanger CRISPR database^26^, cloned into the pSpCas9(BB)-2A-GFP vector, transfected into U2OS (ATCC® HTB-96^TM^) cells using manufacturer-recommended Fugene®HD methodologies, and assayed for knockdown of SHP2 protein levels, via western blot, in bulk. One guide pair against exon 3 of *PTPN11*, 5′-CACCGGATTACTATGACCTGTATGG-3′ and 5′-AAACCCATACAGGTCATAGTAATCC-3′, was observed to achieve knockdown of SHP2 protein levels, and from cells transfected with this construct, single colonies were isolated, assayed for the presence or absence of SHP2 protein, and sequenced from isolated TOPO®-cloned plasmids.

### Design of SHP2 expression plasmids

Mammalian SHP2 expression plasmids were designed from pCMV-SHP2 WT, which was provided as a gift from Ben Neel (Addgene plasmid #8381), using site-directed mutagenesis (QuikChange, Agilent; see Supplementary Table 4 for the sequences of all primers used in this study). Mutation acquisition and plasmid identity were confirmed via DNA sequencing.

### Western blot pErk quantification in U2OS^PTPN11-Null^ cells

1.0 × 10^6^ *PTPN11*−/−/− U2OS (Parental U2OS; ATCC® HTB-96^TM^) cells were plated in six-well tissue culture-treated plates (Corning® CLS3516) in complete growth media two days prior to experiment or 1.0 × 10^6^ U2OS cells were plated 1 day prior to SHP099 addition and allowed to adhere. For *PTPN11*−/−/− U2OS cells, one day prior to addition of SHP099, cells were transfected with respective CMV-driven SHP2 plasmids using manufacturer-recommended FuGENE®HD methodologies, with 0.6 μg DNA and 2.4 μL Fugene®HD per well. On the day of experiment, media was aspirated and replaced with 3 mL media containing either SHP099 at respective concentration or 0.03% dimethyl sulfoxide, and cells were placed at 37 °C with 5% CO_2_ for 2 h. After SHP099 treatment, cells were lysed with cold cell lysis buffer (CST 9803) containing cOmplete^TM^ EDTA-free protease inhibitor cocktail (Roche 11836170001) with PhosSTOP^TM^ (Roche 4906845001) at 4 °C for 30 min. Lysis was visually confirmed with light microscopy, and lysates were added to gel loading buffer and run on Novex^TM^ wedgewell^TM^ 12% Tris-Glycine gels (Invitrogen XP00122) at 180 V for 1 h. Proteins were transferred from gels to polyvinylidene difluoride membranes using an iBlot 2 Dry Blotting System (Thermo Fisher IB21001). After transfer, membranes were blocked with either 5% bovine serum albumin or 5% nonfat milk in tris-buffered saline with tween (TBST) at room temperature for 1 h and then incubated with primary antibody (CST 9101 pErk at 1:500 dilution, CST 9102 Erk at 1:500 dilution, CST 3752 SHP2 at 1:300 dilution, or Sigma A1978 β-Actin at 1:3000 dilution) in respective blocking solution overnight at 4 °C, washed 3 × with TBST for 5 min, incubated with secondary antibody (ECL^TM^ Anti-Rabbit GE NA934V or Anti-Mouse GE NXA931V at 1:10,000 dilution) in respective blocking solution for 1 h at room temperature, and washed 3 x with TBST for 5 min. Following final wash, membranes were imaged on a GE Amersham Imager 600 using SuperSignal® West Pico Chemiluminescent Substrate (Pierce 34080). Linear brightness and contrast adjustments were applied uniformly across the entire image, and band densities were quantified using Fiji^24^. Western blot images are representative of at least two replicates, which were performed on different days.

### Expression and purification of SHP2^E76K^

The gene encoding human SHP2 from residues Met1-Leu525 was inserted into a pET30 vector, and a sequence encoding a 6X histidine tag followed by a tobacco etch virus (TEV) protease cleavage site was added 5′ to the SHP2 gene sequence. The E76K mutation was introduced using an Agilent QuikChange^TM^ Site-Directed Mutagenesis Kit. The resulting construct was transformed into BL21 Star™ (DE3) cells and grown at 37 °C in Terrific Broth containing 100 µg/ml kanamycin. At an OD_600_ of 4.0, SHP2 expression was induced using 1 mM IPTG. Cells were harvested following overnight growth at 18 °C. Cell pellets were resuspended in lysis buffer containing 50 mM Tris-HCl pH 8.5, 25 mM imidazole, 500 mM NaCl, 2.5 mM MgCl_2,_ 1 mM TCEP, 1 ug/ml DNase1, and cOmplete^TM^ EDTA-free protease inhibitor and lysed using a microfluidizer, followed by ultracentrifugation. The supernatant was loaded onto a HisTrap HP chelating column in 50 mM Tris-HCl, 25 mM Imidazole, 500 mM NaCl, 1 mM TCEP, and SHP2^E76K^was eluted with the addition of 250 mM imidazole. The N-terminal histidine tag was removed with an overnight incubation with TEV protease at 4 °C. The protein was subsequently diluted to 50 mM NaCl with 20 mM Tris-HCl pH 8.5 and 1 mM TCEP and applied to a HiTrap Q FastFlow column equilibrated with 20 mM Tris pH 8.5, 50 mM NaCl, and 1 mM TCEP. The protein was eluted with a 10 column-volume gradient, from 50 to 500 mM NaCl. Fractions containing SHP2 were pooled, concentrated, and loaded onto a HiLoad Superdex200 PG 16/100 column to exchange the protein into crystallization buffer (20 mM Tris-HCl pH 8.5, 150 mM NaCl, 3 mM TCEP, and 5% Glycerol). SHP2^E76K^ was concentrated to 10 mg/ml for use in crystallization.

### Crystallization and structure determination

The sitting-drop vapor diffusion method was used for crystallization of both SHP2^E76K^ apo and SHP2^E76K^ in complex with SHP099. SHP2^E76K^ apo was crystallized using a 1:1 volume of protein and well solution (0.1 M MES pH 6.5, 12% polyethylene glycol (PEG) 20,000, and 5% sucrose). Crystals were cryoprotected using the crystallization solution with the addition of 20% glycerol followed by flash freezing directly into liquid nitrogen. SHP2^E76K^ in complex with SHP099 was co-crystallized using a 1:1 volume of protein + 2 mM compound and well solution (0.1 M Tris pH 8.5, 30% PEG 4000, and 0.2 M LiSO_4_). Crystals were cryoprotected using the crystallization solution + 1 mM compound with the addition of 20% glycerol followed by flash freezing directly into liquid nitrogen.

Diffraction data for both SHP2^E76K^ apo and SHP2^E76K^ in complex with SHP099 were collected on a Dectris Pilatus 6 M Detector at beamline 17ID (IMCA-CAT) at the Advanced Photon Source at Argonne National Laboratories. The data were measured from a single crystal maintained at 100°K and exposed to a wavelength of 1 Å, and the reflections were indexed, integrated, and scaled using XDS^27^. The spacegroup of SHP2^E76K^ apo was C2 with two molecules in the asymmetric unit. The structure was determined by molecular replacement using Phaser^28^, with the structure of SHP2^WT^ (PDB: 2SHP) used as a search model. All solvent molecules were removed and the model was divided into its individual domains of N-SH2 (4–104), C-SH2 (112–215) for chain A, and PTP (219–525). The final model was built in the Coot molecular graphics application^29^ and refined using Buster (Global Phasing, Ltd.)^30^ The spacegroup of SHP2^E76K^ in complex with SHP099 was P2_1_, with two molecules in the asymmetric unit. The structure was determined with Fourier methods, using the SHP2^WT^ structure (PDB: 2SHP) with all solvent molecules removed. The final model was built in the Coot molecular graphics application and refined using Buster. Data collection and refinement statistics are shown in Supplementary Table 1.

## Electronic supplementary material

Supplementary Information

## Data Availability

Coordinates and structure factors for the SHP2^E76K^ apoprotein structure and for the SHP099-bound structure of SHP2^E76K^ are deposited in the protein data bank under PDB ID codes 6CRF and 6CRG, respectively. All other source data are available from the corresponding authors on reasonable request and/or are included with the manuscript (as figure source data or Supplementary Information).
