# Supplementary material for: Structural reorganization of SHP2 by oncogenic mutations and implications for oncoprotein resistance to allosteric inhibition
Source: Nat Commun. 2018 Oct 30;9:4508. doi: 10.1038/s41467-018-06823-9 (PMC6207684; doi:10.1038/s41467-018-06823-9)
Supplement: Supplementary file 1 — Supplementary Information [file 41467_2018_6823_MOESM1_ESM.pdf]

Structural reorganization of SHP2 by oncogenic mutations and implications for oncoprotein resistance to allosteric inhibition

J.R. LaRochelle et al. 2018

Supplementary Information

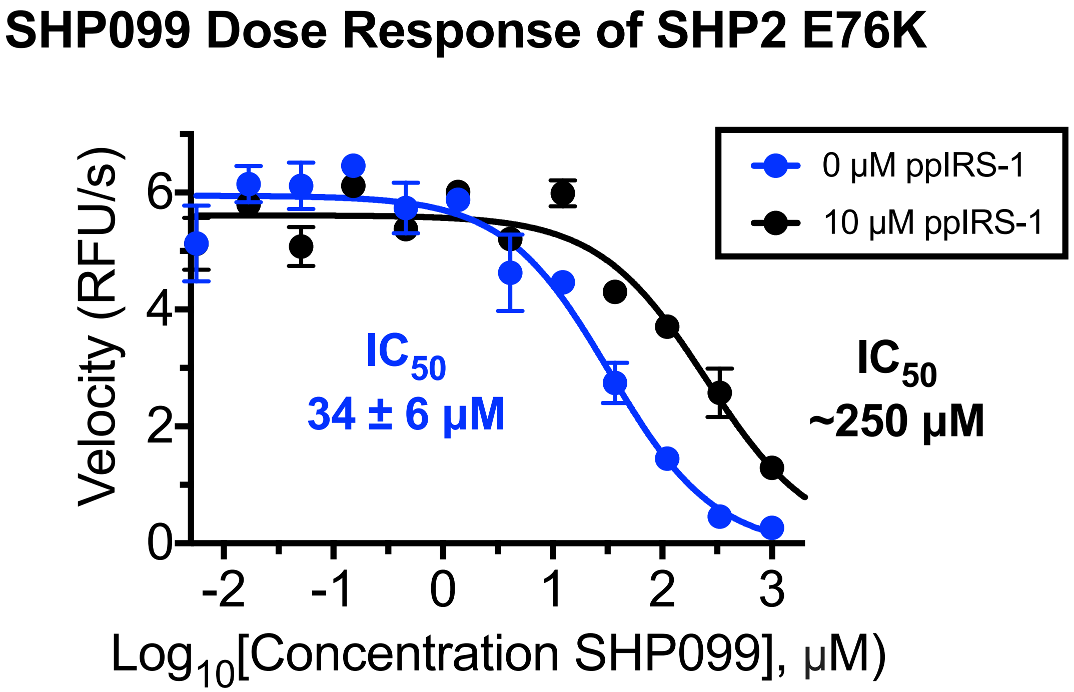

Supplementary Fig. 1. Sensitivity of E76K to inhibition by SHP099 in the absence and presence of phospho-IRS-1. Error bars represent standard error of the mean (SEM) and were calculated using GraphPad Prism.

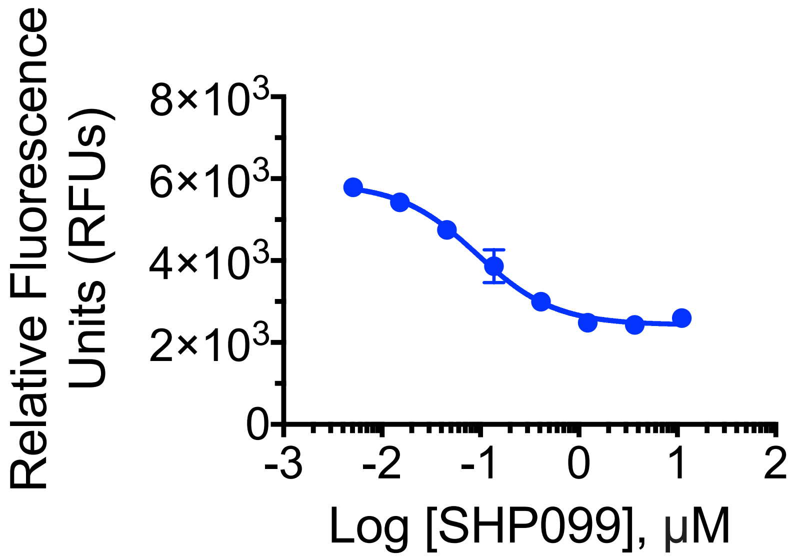

Supplementary Fig. 2. Inhibition of SHP2WT by SHP099 in the presence of 2 nM p-IRS1 bisphosphopeptide. Error bars represent standard error of the mean (SEM) and were calculated using GraphPad Prism.

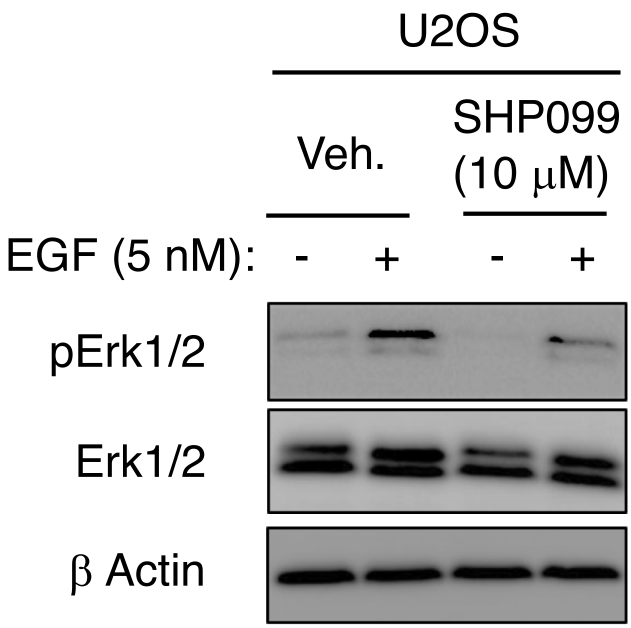

Supplementary Fig. 3. U2OS cells were serum starved for 48 hours, treated with SHP099 or vehicle control for 2 hours, and then stimulated with EGF. Cells were lysed on ice 10 min after stimulation, and pErk levels were analyzed by western blot.

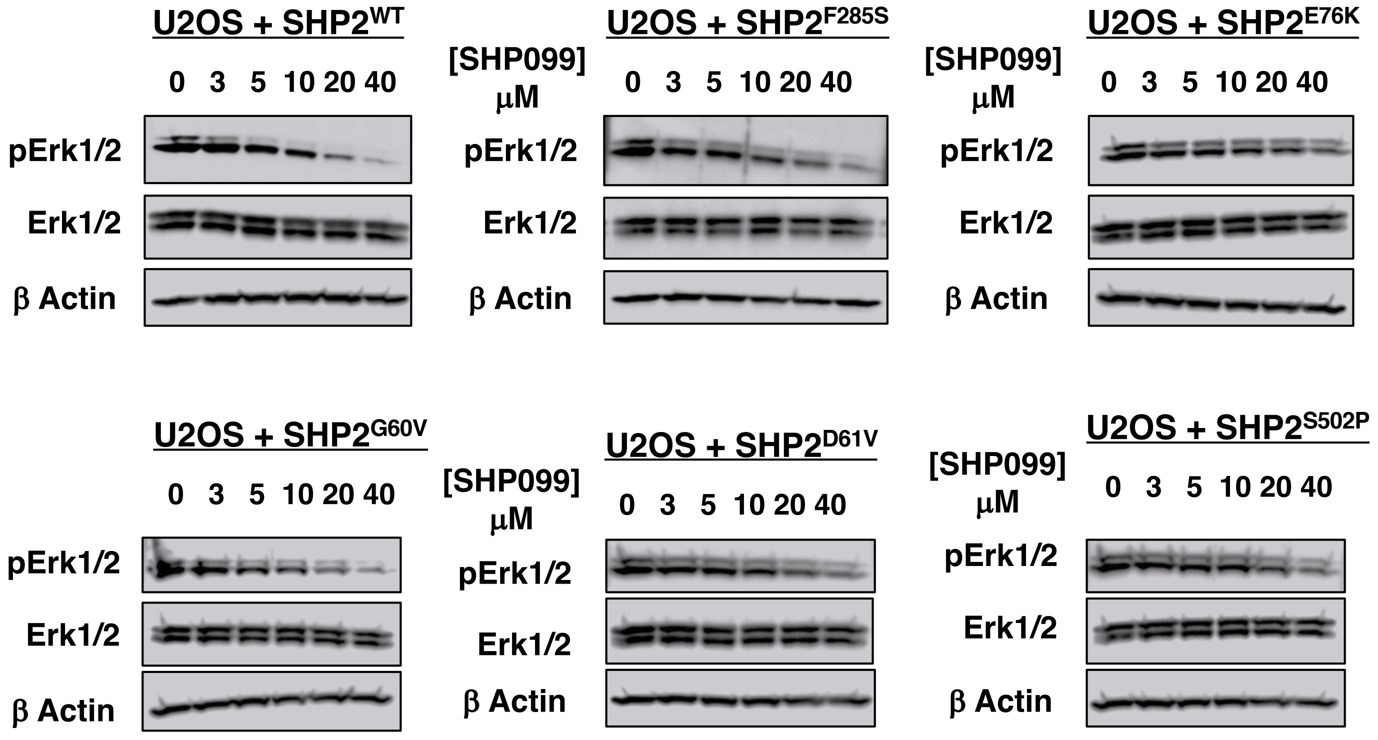

Supplementary Fig. 4. 2.5 x 10^5 U2OS cells were seeded, transfected with various oncogenic forms of SHP2, and treated with SHP099 for 2 hours. pErk levels were analyzed by western blot.

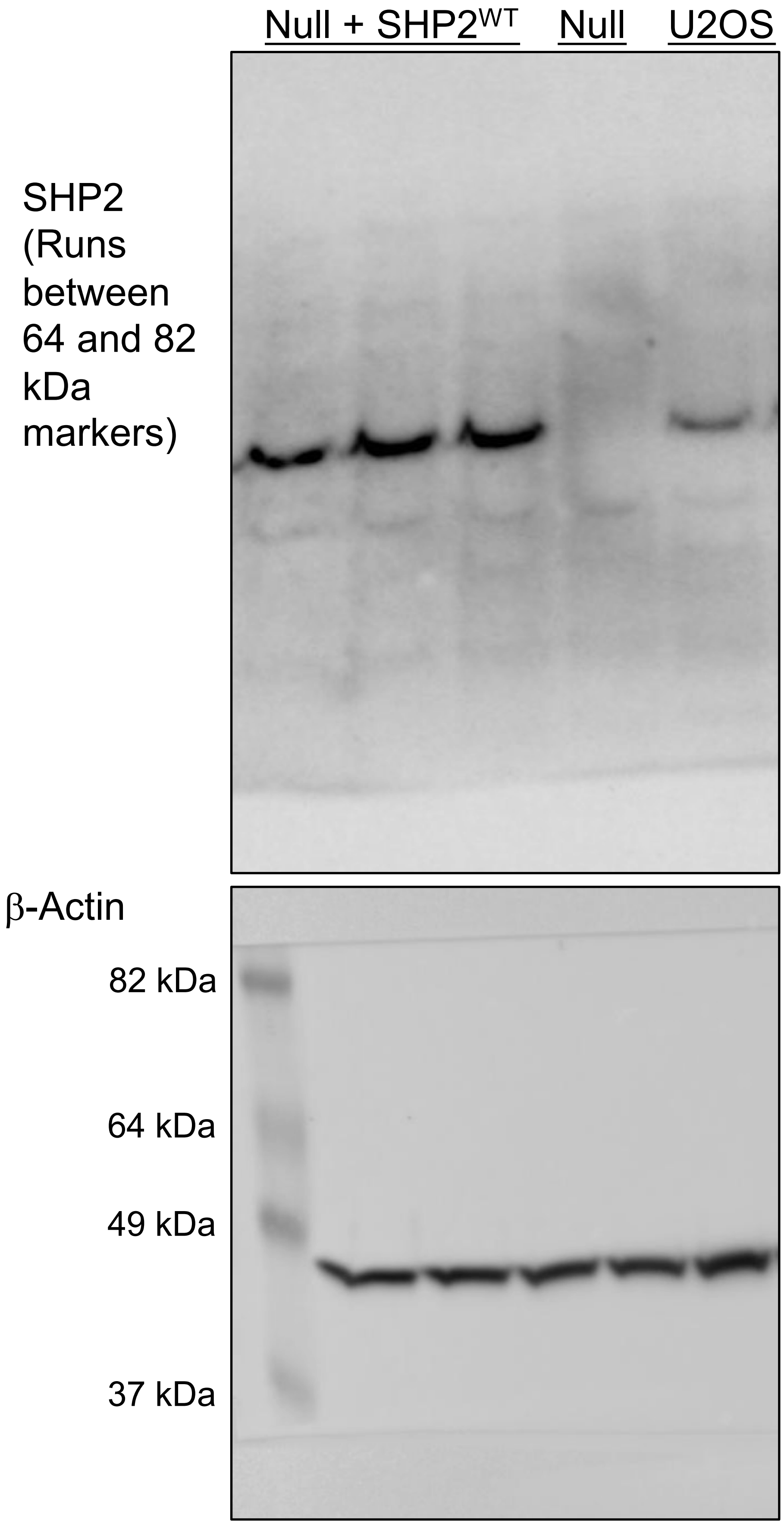

Supplementary Fig. 5. Uncropped lanes from western blots shown in Figure 5B.

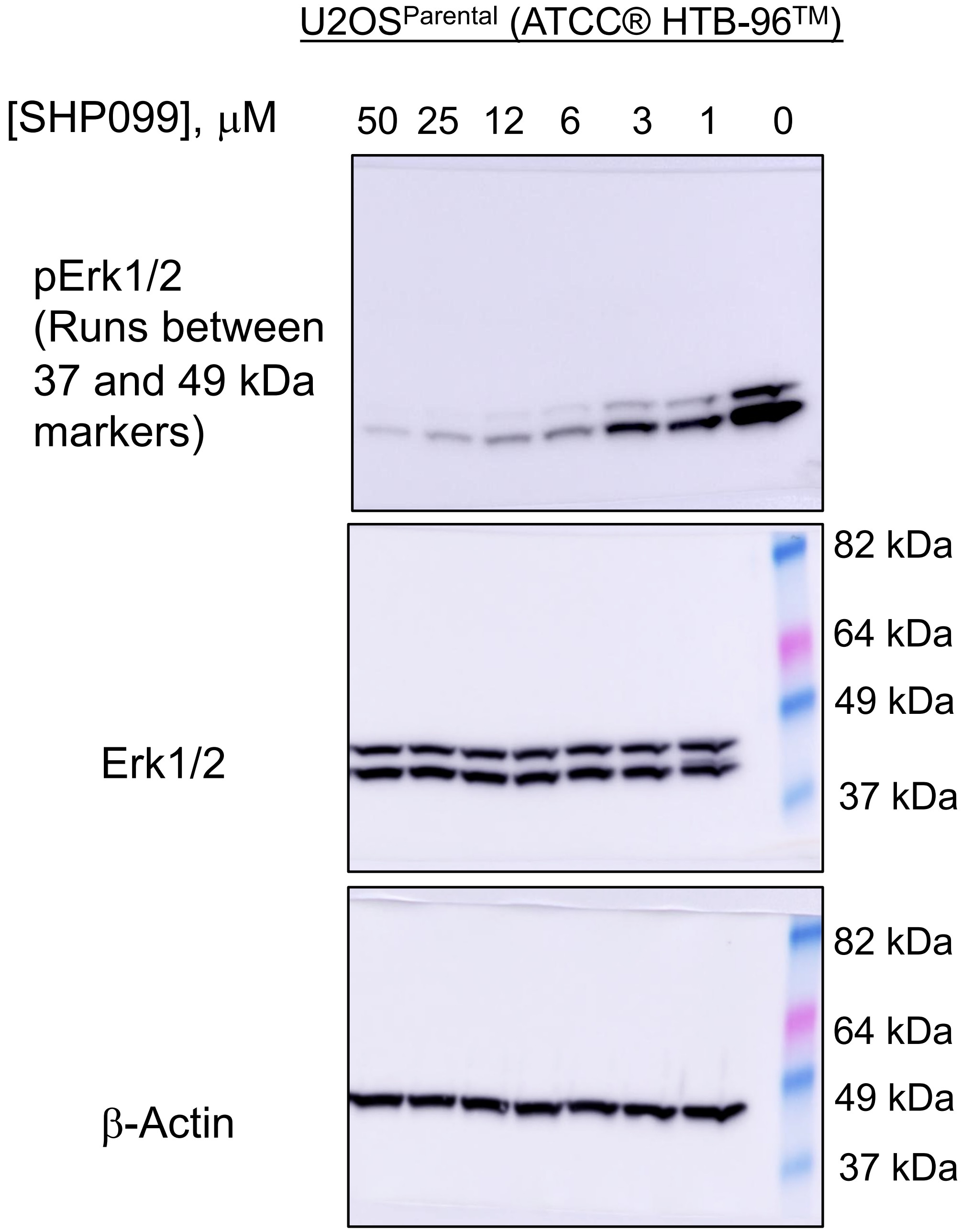

Supplementary Fig. 6. Uncropped lanes from western blots shown in Figure 5C.

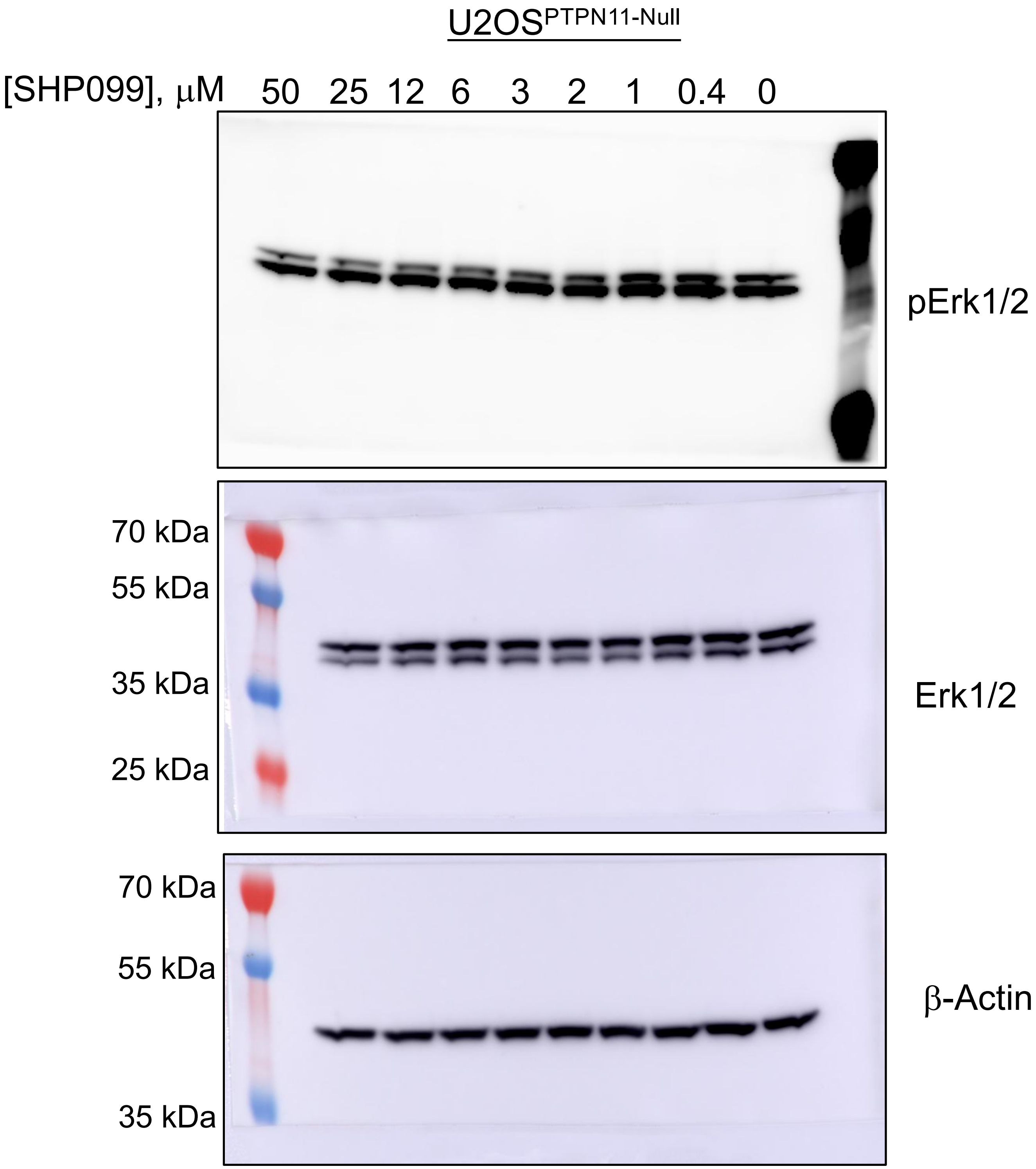

Supplementary Fig. 7. Uncropped lanes from western blots shown in Figure 5D.

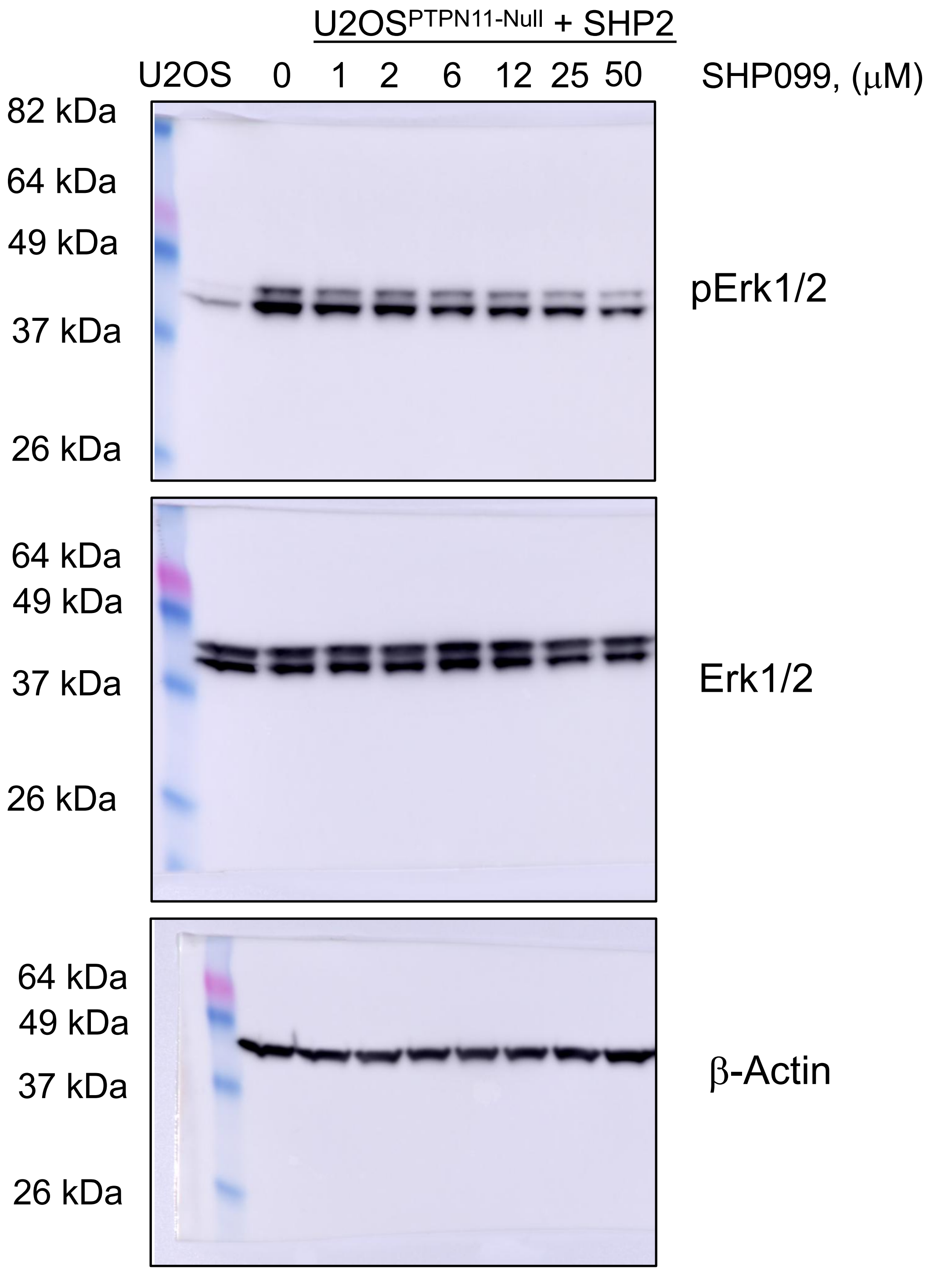

Supplementary Fig. 8. Uncropped lanes from western blots shown in Figure 5E.

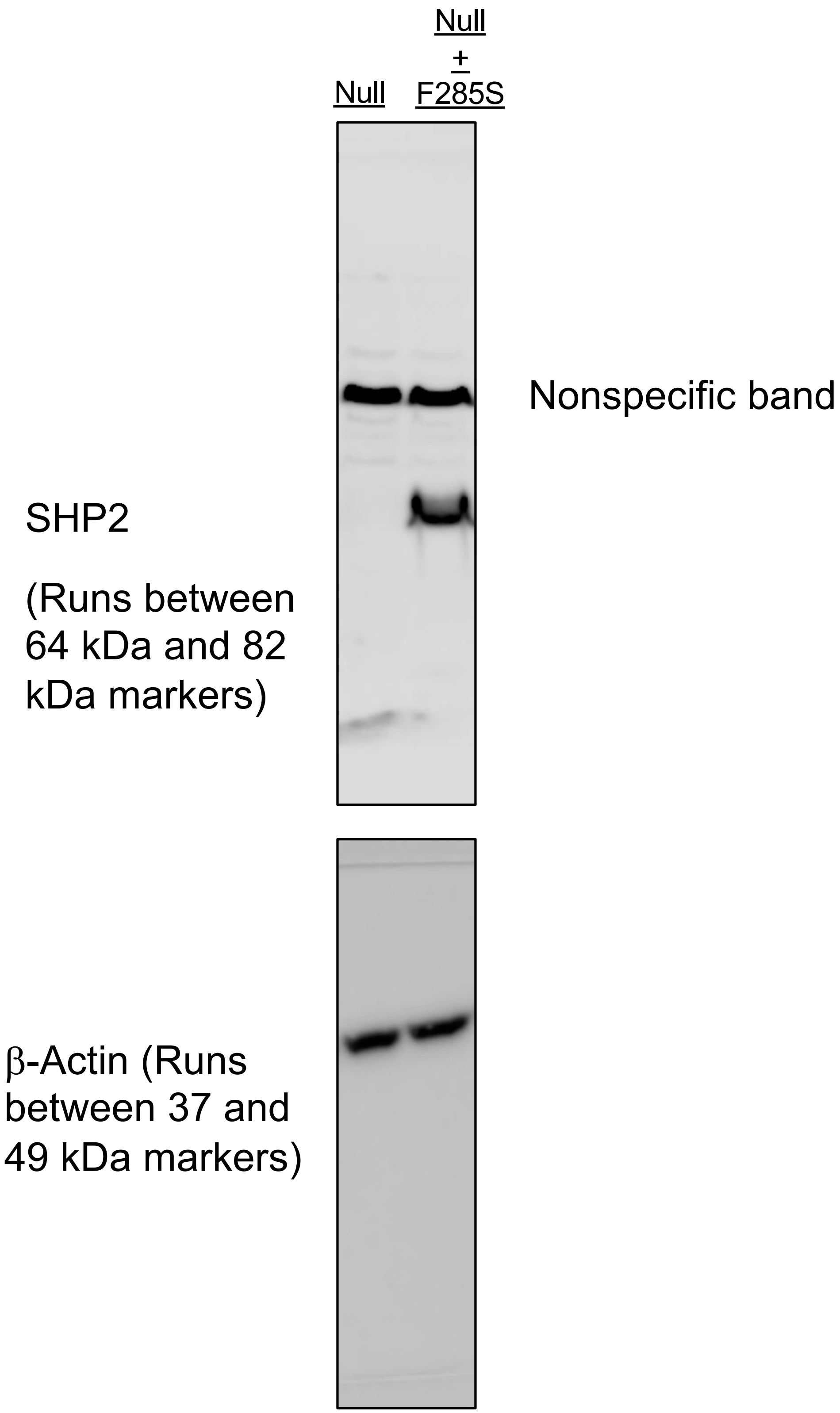

Supplementary Fig. 9. Uncropped lanes from western blots shown in Figure 6A.

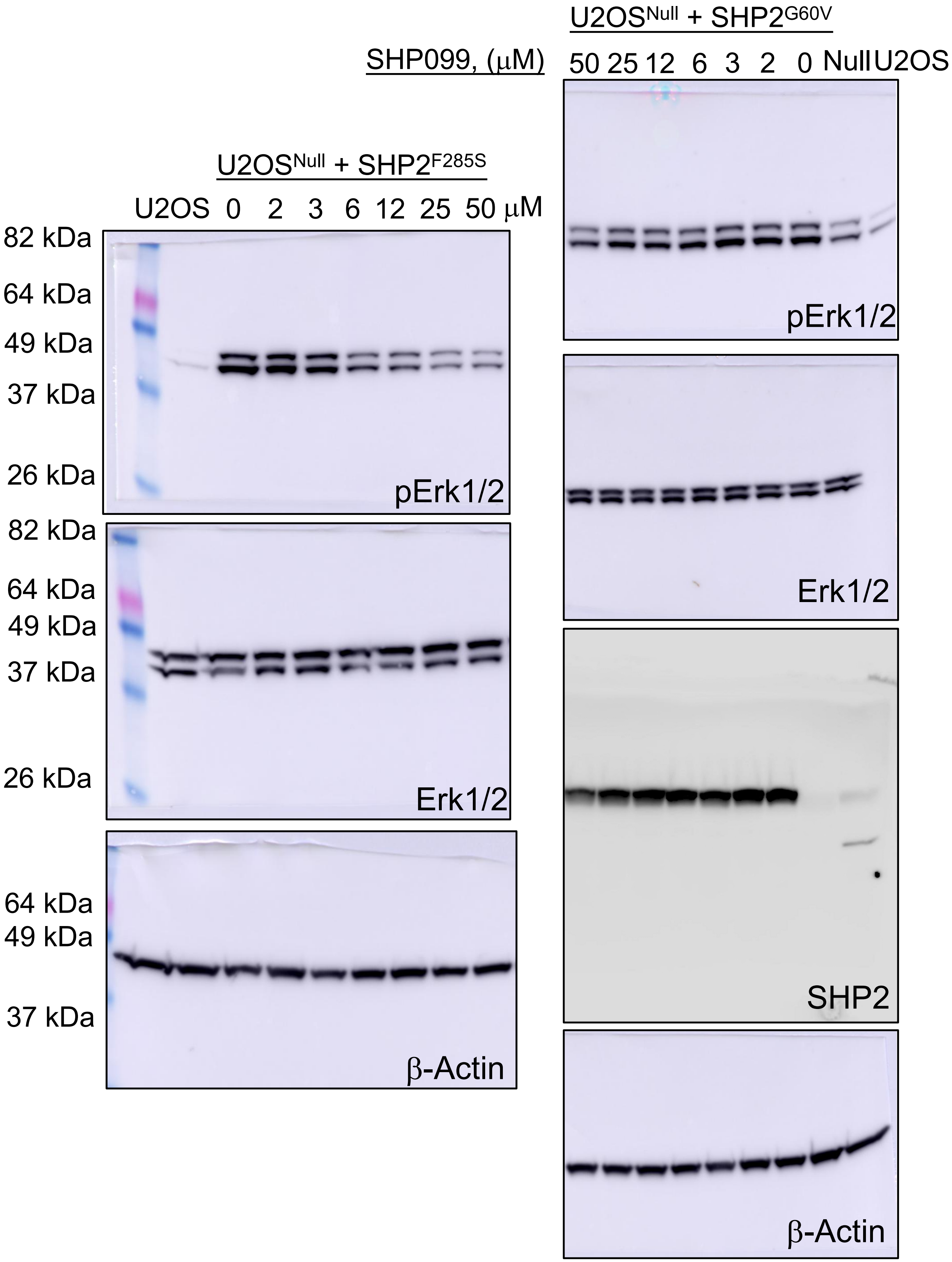

Supplementary Fig. 10. Uncropped lanes from western blots shown in Figure 6A and 6C.

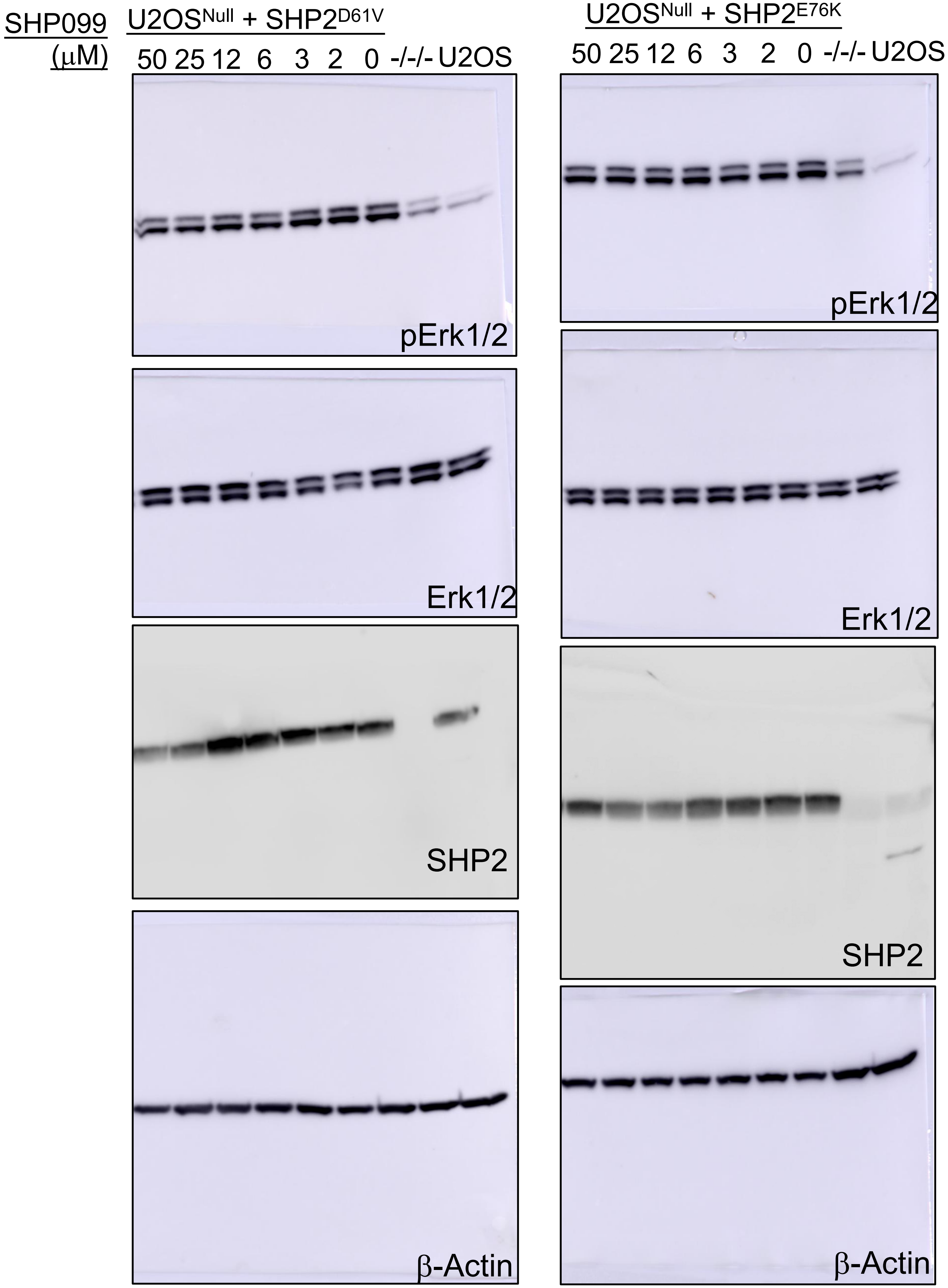

Supplementary Fig. 11. Uncropped lanes from western blots shown in Figure 6A and 6C.

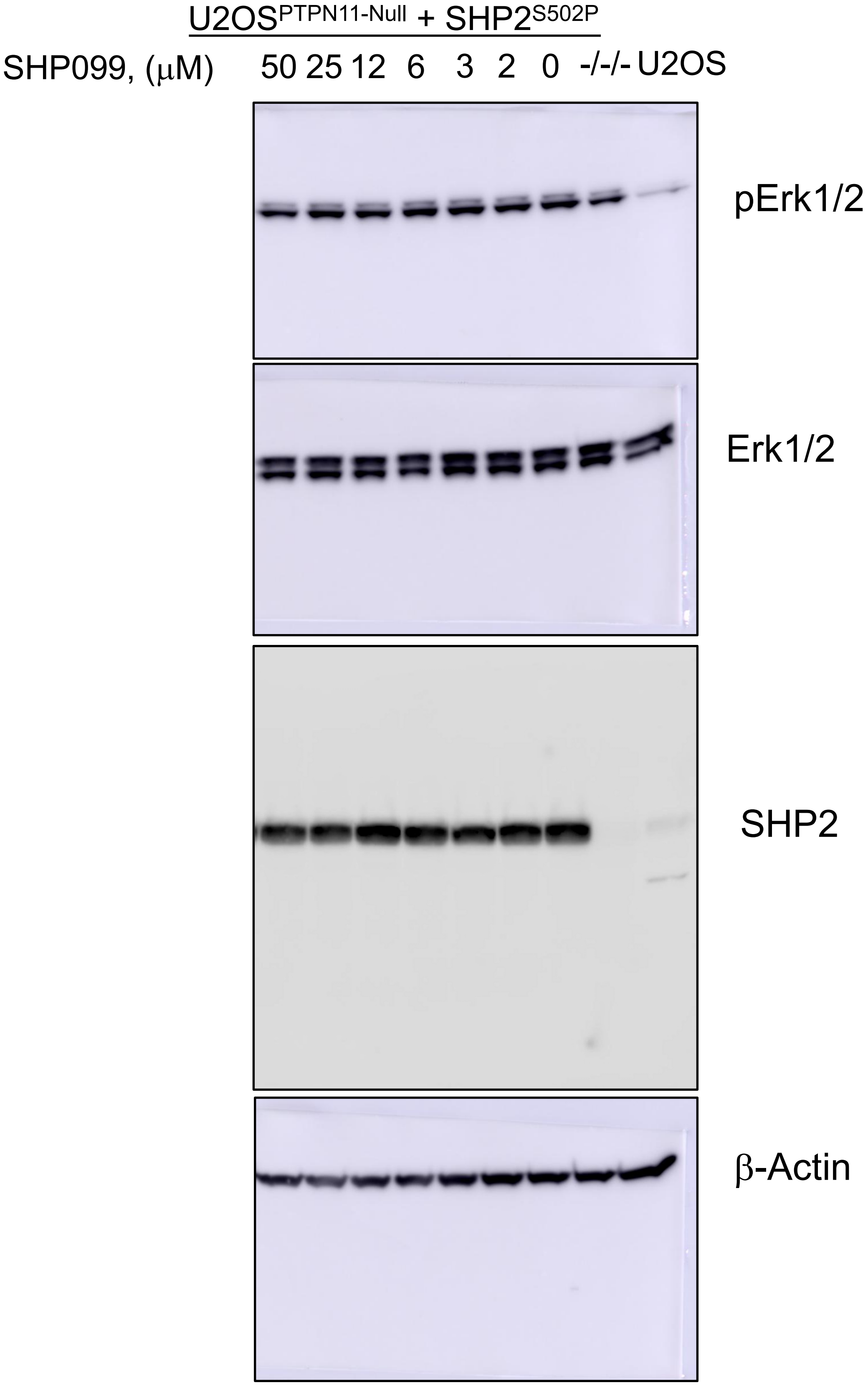

Supplementary Fig. 12. Uncropped lanes from western blots shown in Figure 6A and 6C.

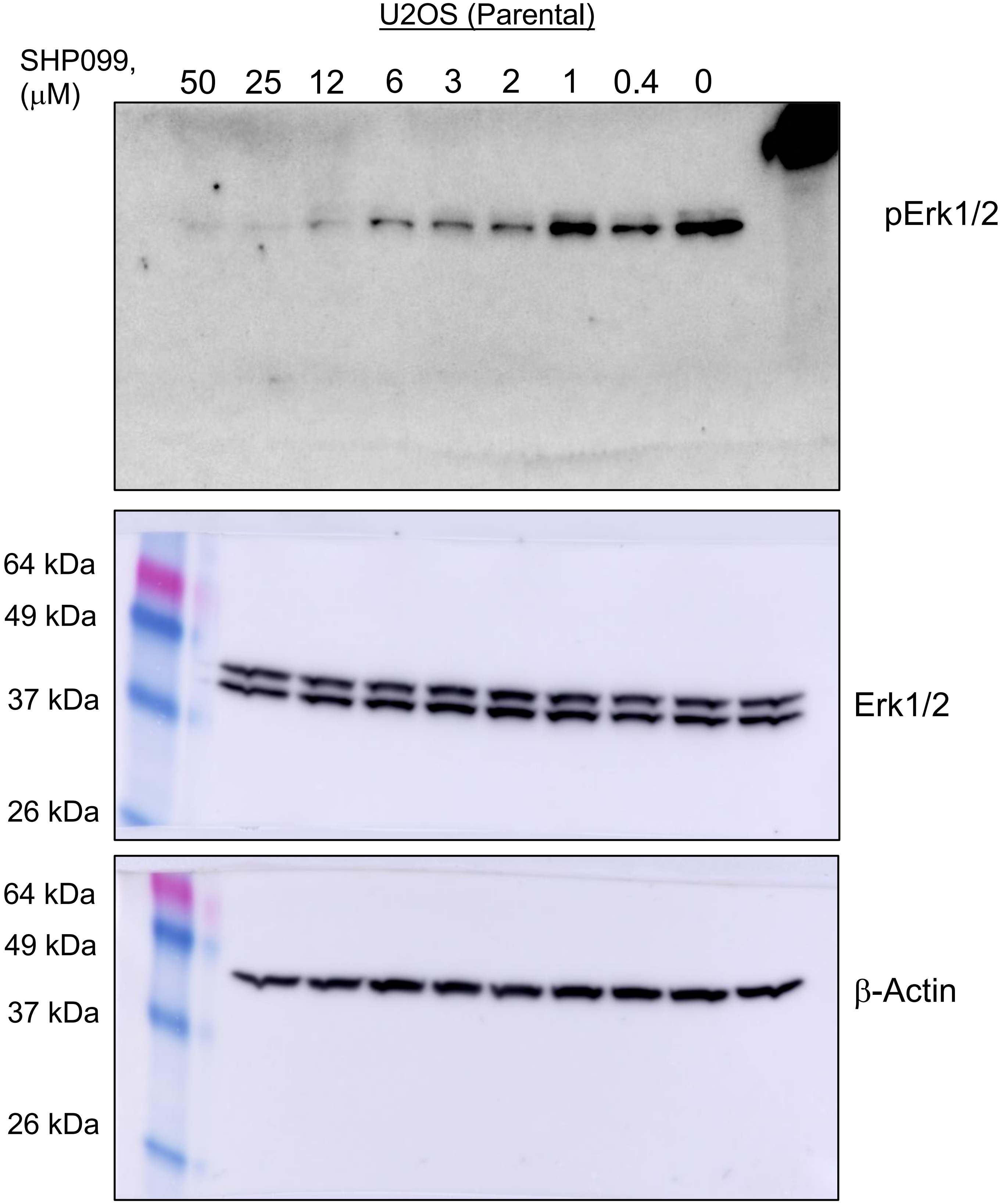

Supplementary Fig. 13. Uncropped lanes of extended SHP099 dose response in U2OS (parental) cells. Data is relevant to Figure 5F.

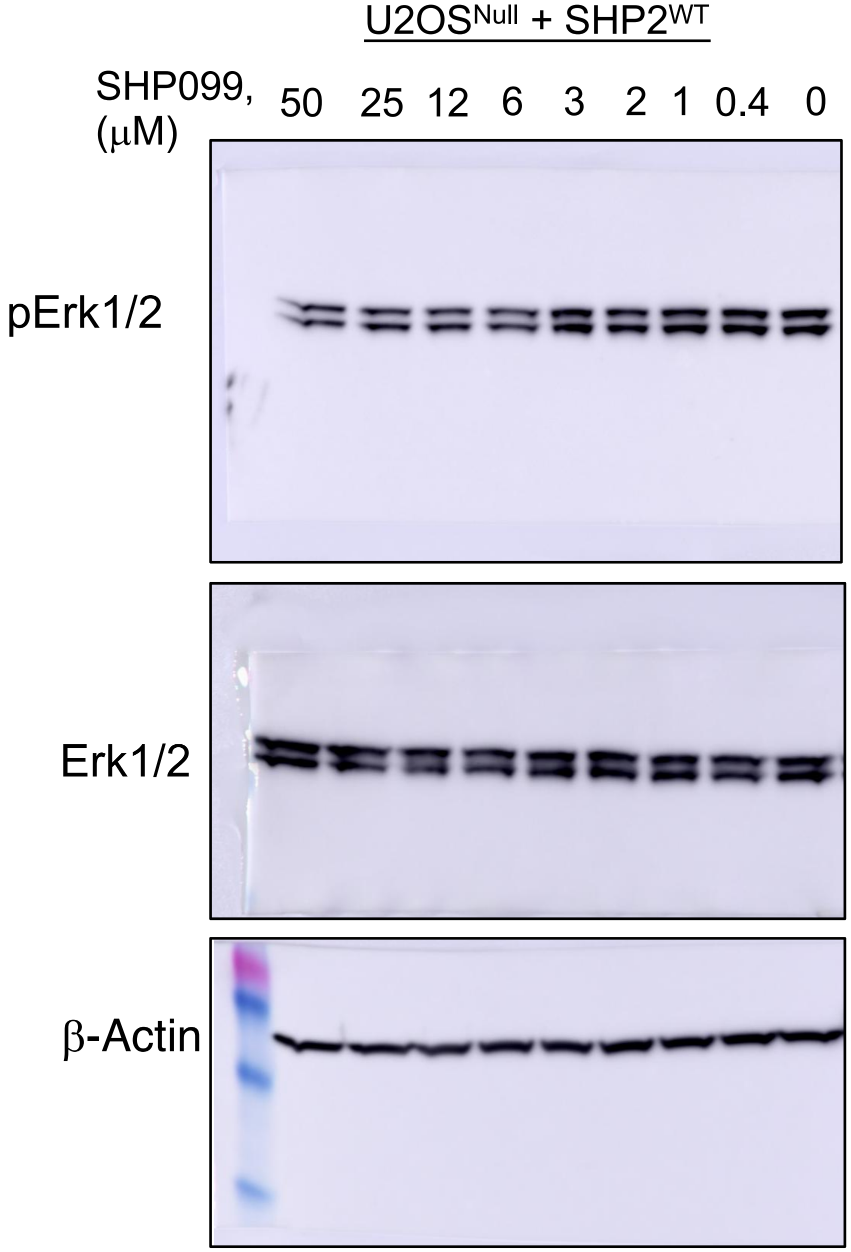

Supplementary Fig. 14. Uncropped lanes of extended SHP099 dose response in U2OS$^{PTPN11\text{-}Null}$ cells re-expressing SHP2 wild-type. Data is relevant to Figures 5F and 6D.

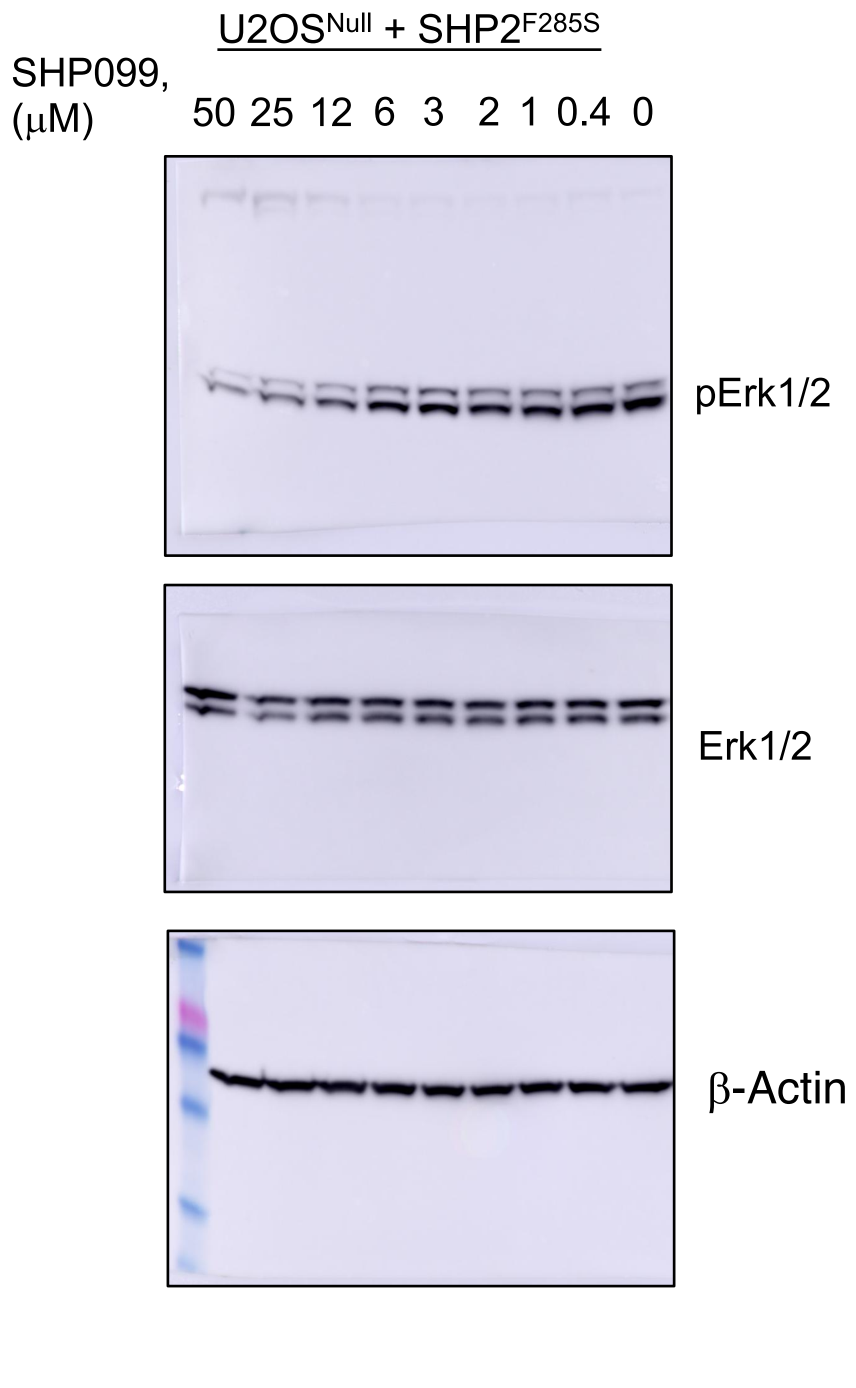

Supplementary Fig. 15. Uncropped lanes of extended SHP099 dose response in U2OS$^{PTPN11\text{-}Null}$ cells re-expressing SHP2^{F285S}. Data is relevant to Figures 5F and 6D.

Supplementary Table 1. Data collection, phasing, and refinement statistics

Parameters	SHP2 E76K	SHP2 E76K · SHP099 Complex
Space group	C2	$P2_1$
Cell dimensions		
a, b, c, Å	249.1, 41.74, 153.9	45.44, 214.1, 55.5
Resolution, Å	124.8-2.62 (2.63-2.62)	107.0-2.75 (2.76-2.75)
R_{merge} [a,b]	6.7 (63.8)	9.1 (60.0)
I/σI [a]	14.5 (2.2)	10.7 (2.1)
Completeness (%) [a]	99.8 (99.5)	99.6 (99.6)
Multiplicity	3.9 (4.0)	3.4 (3.4)
Total Observations	153,455	92,391
Unique reflections	39,327	27,091
R_{work}/R_{free} [c]	19.8 / 23.7 (21.6 / 31.3)	19.6 / 25.0 (23.3 / 32.9)
No. atoms	7,169	7,793
Protein atoms	6,955	7,609
Heterogen Atoms	6	92
Solvent Molecules	208	92
Average B-factor (Å^2)	71.95	63.5
Macromolecules	72.4	63.3
Ligands	-	62.5
Solvent	56.8	50.9
R.m.s deviations		
Bond lengths, Å	0.01	0.01
Bond angles, °	1.14	1.03
Ramachandran Plot (%)		
Favored	94.2	95.4
Allowed	4.41	3.9
Outliers	1.36	0.7

[a] Highest resolution shell is shown in parentheses.

[b] $R_{merge} = \Sigma|I_h - \langle I_h\rangle|/\Sigma I_h$ over all h, where I_h is the intensity of reflection h.

[c] R_{work} and $R_{free} = \Sigma||F_o| - |F_c|| / \Sigma|F_o|$, where F_o and F_c are observed and calculated amplitudes, respectively. R_{free} was calculated using 5% of data excluded from the refinement.

Supplementary Table 2. SAXS-derived radii of gyration (R_g) and maximal end-to-end distances (D_{max}) for $SHP2^{WT}$ and $SHP2^{E76K}$ proteins.

Protein	Guinier, R_g (Å)	Reciprocal Space, R_g (Å)	Dmax (Å)
$SHP2^{WT}$	26.2 ± 0.2	26	83 ± 3
$SHP2^{E76K}$	29.2 ± 0.3	29	100 ± 4

*Errors represent represent standard deviations and were calculated using PRIMUS software.

Supplementary Table 3. Relative activity of SHP2 enzyme variants and sensitivity to inhibition by SHP099 in the absence and presence of p-IRS1.

Protein	Relative Basal Vmax (%)	SHP099 IC_{50}, Basal (μM)	SHP099 IC_{50}, 10 nM pIRS-1 (μM)	SHP099 IC_{50}, 10 μM pIRS-1 (μM)	pIRS-1 AC_{50} (nM)
**$SHP2^{WT}$**	3 ± 1	N.R.	0.11 ± 0.04	1.7 ± 0.13	247 ± 18
**$SHP2^{F285S}$**	20 ± 3	0.068 ± 0.004	0.21 ± 0.05	7.08 ± 1.37	18 ± 2
**$SHP2^{G60V}$**	15 ± 4	0.642 ± 0.058	2.8 ± 0.1	30.2 ± 2.0	9 ± 2
**$SHP2^{S502P}$**	51 ± 2	0.519 ± 0.068	2.6 ± 0.2	102 ± 5	5 ± 2
**$SHP2^{D61V}$**	55 ± 3	0.777 ± 0.126	16 ± 2	>100	4 ± 1
**$SHP2^{E76K}$**	88 ± 7	34 ± 6	> 50	> 400	N.R.
**$SHP2^{PTP}$**	100 ± 8	N.R.	N.R.	N.R.	N.R.

*Errors represent represent standard error of the mean (SEM) and were calculated using GraphPad Prism.

Supplementary Table 4. Sequences of SHP2 Primers Used in this Study.

Primer	Sequence
SHP2_G60V_Forward	GATTCAGAACACTGTTGATTACTATGACCTGTATGGAGG
SHP2_G60V_Reverse	CCTCCATACAGGTCATAGTAATCAACAGTGTTCTGAATC
SHP2_D61V_Forward	CAGAACACTGGTGTTTACTATGACCTGTATGGAGGG
SHP2_D61V_Reverse	CCCTCCATACAGGTCATAGTAAACACCAGTGTTCTG
SHP2_E76K_Forward	GCCACTTTGGCTAAGTTGGTCCAGTATTACATGG
SHP2_E76K_Reverse	CCATGTAATACTGGACCAACTTAGCCAAAGTGGC
SHP2_F285S_Forward	CATCCTGCCCTCTGATCATACCAGGGTTGTC
SHP2_F285S_Reverse	GACAACCCTGGTATGATCAGAGGGCAGGATG
SHP2_S502P_Forward	GGTCTCAGAGGCCAGGGATGGTCCAGAC
SHP2_FS502P_Reverse	GTCTGGACCATCCCTGGCCTCTGAGACC
SHP2_PTP_Forward (for amplification)	ACCTGTATTTTCAGGGATCCGGAGGACGTATAAATGCTGCTGAAAT
SHP2_PTP_Reverse (for amplification)	GCTTTGTTAGCAGCCGGATCCTTATAGTGTTTCAATATAATG
PTPN11_Exon 3_CRISPR_Guide_Forward	CACCGGATTACTATGACCTGTATGG
PTPN11_Exon 3_CRISPR_Guide_Reverse	AAACCCATACAGGTCATAGTAATCC
